# Foliar Application of Silicon Enhances Resistance Against *Phytophthora infestans* Through the ET/JA- and NPR1- Dependent Signaling Pathways in Potato

**DOI:** 10.3389/fpls.2021.609870

**Published:** 2021-01-28

**Authors:** Xiaojing Xue, Tiantian Geng, Haifeng Liu, Wei Yang, Weiran Zhong, Zhiliang Zhang, Changxiang Zhu, Zhaohui Chu

**Affiliations:** ^1^State Key Laboratory of Crop Biology, College of Agronomy, Shandong Agricultural University, Tai’an, China; ^2^Key Laboratory of Quality Improvement of Agricultural Products of Zhejiang Province, School of Agriculture and Food Science, Zhejiang A&F University, Hangzhou, China; ^3^Institute of Characteristics Crops, Chongqing Academy of Agricultural Sciences, Chongqing, China

**Keywords:** defense response, ethylene, late blight, jasmonic acid, silicon, signaling pathway

## Abstract

Late blight (LB), caused by the oomycete pathogen *Phytophthora infestans*, is a devastating disease of potato that is necessary to control by regularly treatment with fungicides. Silicon (Si) has been used to enhance plant resistance against a broad range of bacterial and fungal pathogens; however, the enhanced LB resistance and the molecular mechanisms involving the plant hormone pathways remain unclear. In this study, Si treatment of potato plants was found to enhance LB resistance in both detached leaves and living plants accompanied by induction of reactive oxygen species (ROS) production and pathogenesis-related genes expression. Regarding the hormone pathways involved in Si-mediated LB resistance, we found a rapidly increased content of ethylene (ET) 15 min after spraying with Si. Increased jasmonic acid (JA) and JA-Ile and decreased salicylic acid (SA) were identified in plants at 1 day after spraying with Si and an additional 1 day after *P. infestans* EC1 infection. Furthermore, pretreatment with Me-JA enhanced resistance to EC1, while pretreatment with DIECA, an inhibitor of JA synthesis, enhanced the susceptibility and attenuated the Si-mediated resistance to LB. Consistent with these hormonal alterations, Si-mediated LB resistance was significantly attenuated in *StETR1*-, *StEIN2*-, *StAOS*-, *StOPR3*-, *StNPR1*-, and *StHSP90-*repressed plants but not in *StCOI1-* and *StSID2-*repressed plants using virus-induced gene silencing (VIGS). The Si-mediated accumulation of JA/JA-Ile was significantly attenuated in *StETR1*-, *StEIN2*-, *StOPR3-* and *StHSP90*-*VIGS* plants but not in *StCOI1*-, *StSID2-* and *StNPR1-VIGS* plants. Overall, we reveal that Si can be used as a putative alternative to fungicides to control LB, and conclude that Si-mediated LB resistance is dependent on the ET/JA-signaling pathways in a *StHSP90-* and *StNPR1-*dependent manner.

## Introduction

Silicon (Si) is the second most abundant element in the earth’s crust (Brackhage et al., 2013). It is not an essential plant nutrient; however, an increasing number of studies have demonstrated that it is a beneficial substance extensively used in agricultural systems to help plant adaptation to changeable environmental circumstances. The impressive beneficial effects of Si alleviate the destruction of various plant species during abiotic and biotic stresses (Li et al., 2018; Coskun et al., 2019). Si fertilization has been used to alleviate a wide range of abiotic stresses, including UV-B radiation (Shen et al., 2010), extreme temperature (Muneer et al., 2017), salinity (Lee et al., 2010), drought (Coskun et al., 2016), and heavy metal toxicity (Kim et al., 2014). Si application is also beneficial by enhancing plant resistance to biotic stresses. It enhances plant resistance to insect herbivores, such as caterpillar *Cnaphalocrocis medinalis* (rice leaf folder) in rice and the borer *Eldana saccharina* in sugarcane (Reynolds et al., 2009). Si application also has positive effects against pathogens, including viruses, bacteria, fungi and oomycetes in thirty-eight plant species (Wang et al., 2017). Particularly, the preventative function of Si is overwhelmingly associated with pathogens (e.g., powdery mildews, oomycetes and rice blast fungus) that have a biotrophic phase (Kim et al., 2002; Rémus-Borel et al., 2005; Rasoolizadeh et al., 2018), while it has no effect or a negative effect on some typical necrotrophs, such as *Botrytis cinerea* and *Sclerotinia sclerotiorum* (Heine et al., 2006; Nascimento et al., 2014; Coskun et al., 2019).

The roles of Si in higher plants have remained a quandary. In essence, the differential absorption and the mechanism(s) by which Si confers protection against biotic and abiotic stresses are still puzzling. Si accumulation in the above ground part varies greatly with plant species, ranging from 0.1 to >10% of dry weight (Hodson et al., 2005). Three mechanisms, active, passive and rejective, have been described and are associated with high-, intermediate- and low-accumulator plants. Uptake of Si (Si(OH)_4_) in the roots is mediated by specific influx channels (termed Lsi1) and efflux transporters (termed Lsi2). The location and activity of these two influx transporters partially explains the differential Si absorption. In addition to being related to Si accumulation, Lsi1 is involved in Si-mediated biotic stresses (Vivancos et al., 2015; Lin et al., 2019). Si has been associated with the priming of the plant induced systemic resistance (ISR) in recent years. Since then, the link between Si feeding and ISR, and the priming role of Si has been shown in numerous plant-pathogen and plant-insect interactions. The mechanisms by which Si protects plants against pathogens are mainly comprised of physical (Sun et al., 2010), biochemical and molecular aspects, involving the strength of the cell wall and the formation of papillae, increasing the activity of defense-related enzymes (Datnoff et al., 2007), stimulating the production of antimicrobial compounds, activating the expression of defense-related genes, and regulating the hormone signaling pathways, such as salicylic acid (SA), jasmonic acid (JA), and ethylene (ET) (Malamy et al., 1990; Lund et al., 1998; Vijayan et al., 1998; Ye et al., 2013; Kim et al., 2014). In other cases, Si application can modulate plant volatile emissions to enhance the attraction of a pest’s natural enemies (Kvedaras et al., 2010). In addition, it was observed to interfere with effector-receptor expression during the *Phytophthora sojae*-soybean interaction, by enhancing the expression of resistance genes in the host as well as repressing the effector expression in *P. sojae* (Rasoolizadeh et al., 2018).

The phytohormones SA, JA, and ET play key roles in regulating plant defense responses to biotic stresses. In the model plant *Arabidopsis*, SA is mainly responsible for resistance against biotrophic and hemibiotrophic pathogens (Summermatter et al., 1995), whereas JA and ET are mostly involved in resistance against necrotrophic pathogens (Vijayan et al., 1998). SA and JA usually manifested as an antagonistic effect in plants. Si application can induce the expression of a large spectrum of hormone-related genes, such as JA-related genes in anti-herbivore defense (Ye et al., 2013; Lin et al., 2019), SA- and JA-related genes in resistance against *P. sojae* in soybean (Rasoolizadeh et al., 2018), and JA-and SA-/ET-related genes in resistance against *Ralstonia solanacearum* in tomato (Ghareeb et al., 2011; Qiu et al., 2016; Jiang et al., 2019). Accumulated Si also manipulated the signaling pathway of SA, JA and ET (Ye et al., 2013; Vivancos et al., 2015; Jang et al., 2018; Jiang et al., 2019). In *Arabidopsis*, Si treatment resulted in accumulation of SA and enhanced resistance against powdery mildew. However, the resistance is maintained in SA-deficient mutants of *pad4* and *sid2*, indicating that the Si-mediated resistance against powdery mildew is independent of the SA signaling pathway (Vivancos et al., 2015). In the tomato-*R. solanacearum* interaction, Si application was accompanied by accumulation of JA and reduction of SA and ET (Jiang et al., 2019). Si-enhanced resistance to insect herbivory was required for accumulation of JA and function of the JA signaling pathway in rice (Ye et al., 2013). In addition to increasing accumulation of JA, Si application increased the level of gibberellin (GA_1_) and SA (Jang et al., 2018). In the rice-*Cochliobolus miyabeanus* pathosystems, Si-mediated resistance was associated with repressed synthesis of ET for both host and pathogens that is also dependent on the ET signaling pathway (van Bockhaven et al., 2015). However, the cross-talk between ET/JA and SA in Si-mediated resistance remains unclear. Previously, the typical microbe-associated molecular patterns (MAMPs) and copper ion (Cu^2+^)-triggered plant immunity had been identified to initiate from ET to SA (Durrant and Dong, 2004; Zipfel et al., 2006; Liu et al., 2015, 2020; Zhang et al., 2018). PAMPs such as chitosan, chitin, β-1,3-glucan, Flg22 and Nep1 were also involved in JA/ET and SA signaling pathways (Doares et al., 1995; Klarzynski et al., 2000; Bae et al., 2006; Denoux et al., 2008; Lehtonen et al., 2012). Several biocontrol microorganisms mediated ISR is dependent on the JA/ET and SA signaling pathways. For instance, the rhizobacterium *Pseudomonas fluorescens* WCS417r and *Bacillus cereus* AR156 triggered ISR against *Pseudomonas syringe* pv. *Tomato* (*Pst*) DC3000 and *B. cinerea* in *Arabidopsis*, respectively. These forms of ISR are independent of the SA signaling pathway and instead act in a JA/ET- and *NPR1*-dependent manner (Pieterse et al., 2002; Nie et al., 2017).

Potato (*Solanum tuberosum* L.) is the third most important food crop for human consumption in the world after rice and wheat. Late blight (LB), caused by the oomycete pathogen *P. infestans*, is considered the most devastating disease in potato production (Kamoun et al., 2015). There is no report that Si application can enhance the resistance of potato against *P. infestans*. However, in field practice, we observed that a farmer had successfully used the foliar application of Si to control LB on potato. Alternatively, most Si application was conducted as a component of fertilizer because it can be absorbed by the influx transporter Lsi1 located on roots. Only a few studies reported that foliar treatment of Si has an effect (Rodrigues et al., 2010; Assis et al., 2013). Thus, it is of value to investigate the specific mechanisms of Si-mediated resistance against *P. infestans*. In this study, we demonstrated that foliar treatment of Si protected potato from *P. infestans* infection. Si activated potato immunity as well as induced the accumulation of ET and JA. By repressing the expression of ET, SA, and JA synthesis- or signal transduction-related genes via VIGS, we reveal that Si mediated resistance against *P. infestans* through the ET/JA- and NPR1-dependent signaling pathways in potato.

## Materials and Methods

### Bacterial Strains, Oomycete Strains and Plasmids

The bacterial strains, oomycete strains and plasmids used in this study are described in Supplementary Table 1. The *Escherichia coli* strains were cultured on Luria-Bertani (LB) medium at 37°C. The *Agrobacterium tumefaciens* strains were cultured on LB medium containing 50 μg mL^–1^ rifampicin at 28°C. The *P. infestans* EC1 were cultured on Rye A medium at 18°C (Caten and Jinks, 1968).

To knockdown the target genes in potato, virus-induced gene silencing (VIGS) was performed according to the described TRV-EIN2 (Ratcliff et al., 2001; Liu et al., 2020). Approximately 200 bp DNA fragments of each gene were amplified using primers as described in Supplementary Table 2, and inserted into the vector pTV00 (Ratcliff et al., 2001). Each construct was validated by DNA sequencing.

### Plant Materials and Chemical Treatments

Potato plants of variety Désirée were grown in nutrient substrates in a climate chamber with 12-h days (at 23°C) and 12-h nights (at 21°C) at 60–75% relative humidity. Na_2_SiO_3_ (100 mM, pH = 10, hereafter named Si) was dissolved in ddH_2_O supplemented with 0.05% (V/V) Tween 20 and sprayed on potato leaves, with ddH_2_O supplemented with 0.05% (V/V) Tween 20 (Si−) as the control. Methyl jasmonate (100 μM) or the jasmonic acid biosynthesis inhibitor sodium diethyldithiocarbamate (DIECA, 100 μM) were dissolved in ddH_2_O supplemented with 0.05% (V/V) Tween 20 (Farmer et al., 1994). They were sprayed on potato leaves separately or mixed with Si.

### The Growth Inhibition of *P. infestans* EC1

The mycelia disks (7-mm diameter) taken from the cultured *P. infestans* EC1(Race: 2.4.10.11, kindly provided by Professor Tian from Huazhong Agricultural University) plate were transferred to Rye A medium containing 100 mM Na_2_SiO_3_ (Si+) and incubated at 18°C. The growth of EC1 was analyzed by measuring the diameter of the colony 7 days after incubation. All experiments were repeated three times.

### Inoculation, Assessment and Disease Index

Pathogen inoculation was normally performed at 24 h following Si treatment. As described previously (Vleeshouwers et al., 1999; Wang et al., 2019), the sporangia were collected from the cultured *P. infestans* EC1 plate by flooding with ice-cold water. Subsequently, it was incubated in an ice-water bath for an additional 2 h to release zoospores and adjusted to 50,000 mL^–1^ with distilled water. The detached leaves were inoculated with 10 μL of droplet on each side of the leaves, placed in sealed boxes and kept in weak light at 21°C for 3–5 days. A living plant assay was conducted in the chamber for pot experiments. An optically clear plastic cover was put on the container to keep the humidity after spraying inoculation. Disease indexes were measured on approximately 24 leaves for each treatment and scored as follows: “0” no visible infection; “1” < 25% infection; “2” 26–50% infection; “3” 51–75% infection; “4” 76–100% infection. The disease index = $\sum_{0}^{4}x\,i\,y\,i$/(x max ∑*yi*) × 100% (xi represents different scores, and yi represents the number of leaves belonging to different scores). The cell death caused by EC1 was evaluated by staining with trypan blue. The growth of hyphae in potato leaves was quantified by measuring the biomass of *P. infestans-*specific *PiO8* element using qPCR as described previously (Judelson and Tooley, 2000; Liu et al., 2020).

### Histochemical Detection of Superoxide and Hydrogen Peroxide

The accumulation of superoxide and hydrogen peroxide were assessed using nitro blue tetrazolium (NBT) and 3,3′-diaminobenzidine (DAB) staining as described previously (Wohlgemuth et al., 2002; Liu et al., 2015). Detached leaves were inoculated with EC1 by spraying with a suspension of zoospores at 24 h following Si treatment. The samples were harvested at different time points (3, 6, and 12 h) post-treatment with Si and inoculation with *P. infestans* EC1 at 0, 1, and 2 dpi. Water supplemented with 0.05% (V/V) Tween 20 was used as the control.

### RNA Extraction and Quantitative RT-PCR

Total RNA was extracted from leaves using a Plant RNA Kit (Omega Bio-tek, United States) as the manufacturer’s instructions. First-strand cDNA was synthesized using the ReverTra Ace qPCR RT Master Mix with the gDNA Remover kit (TOYOBO, Japan). Quantitative real-time PCR (qRT-PCR) was performed on a qTOWER^3^G Touch thermal cycler (Analytik Jena, Germany) with Ultra SYBR Mixture (with ROX) (CWBIO, China) as described in the manufacturer’s instructions. The PCR program was performed as described previously (Yang et al., 2019; Liu et al., 2020). The *StEF1* (LOC102600107) was used as an internal control to normalize the results. Real-time PCR (RT-qPCR) data were transformed into log2-delta CT for one-way ANOVA followed by Tukey’s test analysis (*P* < 0.05). All qRT-PCR experiments were repeated at least twice in triplicate. The primers used in qRT-PCR are listed in Supplementary Table 2.

### Virus-Induced Gene Silencing in Potato

Virus-induced gene silencing was performed on potato variety Désirée as previously described (Brigneti et al., 2004; Liu et al., 2020). *A. tumefaciens* strain GV3101 carrying pTRV1 (RNA1) and the various pTV00 (RNA2) plasmids were mixed in a 1:1 ratio to co-infiltrate into 3-week-old potato plants by vacuum. The silence effects were predicted with the parallel control of pTRV2-StPDS, which showed signs of bleach on new leaves, and confirmed by qRT-PCR at 4 weeks after agro-infiltration.

### Plant Hormone Measurements

To measure the level of ET, potato plants were sprayed with 100 mM Si or ddH_2_O supplemented with 0.05% (V/V) Tween 20. The ET contents were measured by gas chromatography as described previously (Zhang et al., 2018; Liu et al., 2020). For quantifying the content of SA, JA, and JA-Ile, the leaves were ground in liquid nitrogen, and each of the 100 mg samples was prepared and tested using an HPLC-MS/MS system as previously reported (Xu et al., 2016; Wang et al., 2019). At least three biological replicates were analyzed.

### Statistical Analysis

All data analyses were repeated three times with three replicate experiments independently. Standard deviations were indicated by error bars. Values were analyzed by the GraphPad Prism software package^1^ and the level of significance was determinate using one-way analysis of variance (ANOVA) followed by Tukey’s multiple comparisons test. The mean differences were also compared using Student’s *t-*test.

## Results

### Foliar Treatment of Si Enhances Potato Resistance Against *Phytophthora infestans*

**FIGURE 1:**
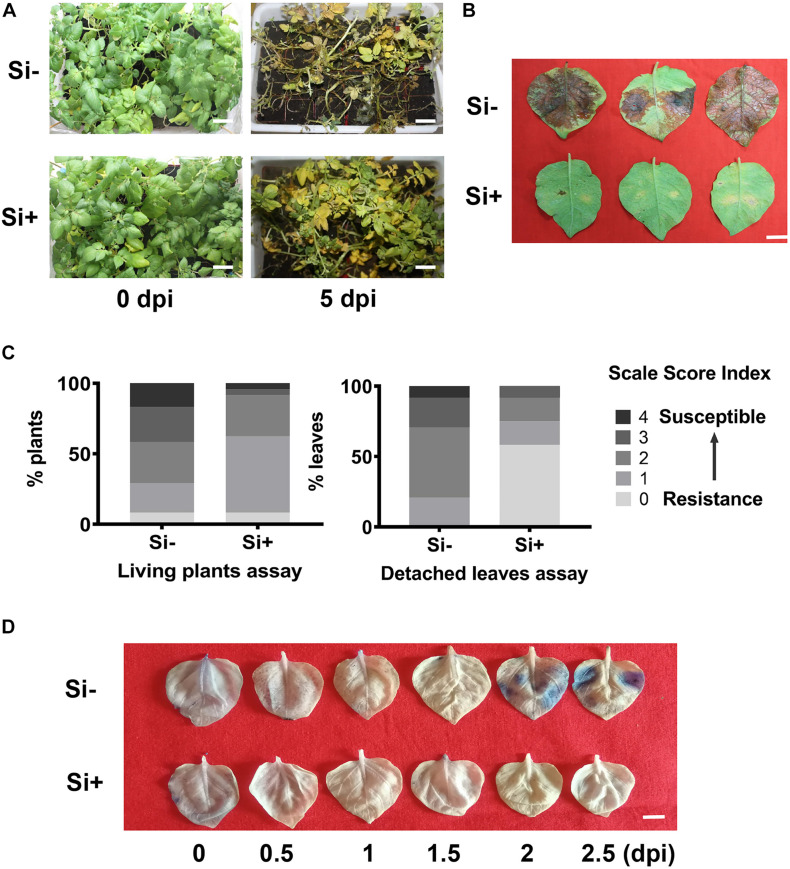
Foliar spraying with Si enhances potato resistance to late blight caused by *Phytophthora infestans*. Disease symptoms of living plants assay in the pot experiment at 5 days post inoculation **(A)** and detached leaves assay at 3 dpi. Scale bar represents 5 cm. **(B)** The plants or leaves were inoculated with *P. infestans* EC1 at 24 h following spraying the 100 mM sodium silicate (Si) on leave. Scale bar represents 1 cm. **(C)** Disease index of living plants assay and detached leaves. More than 24 plants were performed in each pot experiments assay. And 30 leaves from 10 individual plants were inoculated in each detached leave assay. **(D)** Infected leaves were stained with trypan blue at different time points. Three leaves were randomly selected from 12 plants at 0, 0.5, 1.0, 1.5, 2.0, and 2.5 days post inoculation EC1 following Si treatment. Three independent experiments were performed for above assays with similar results. Scale bar represents 1 cm.

Si fertilizer has been widely used to enhance plant resistance to different abiotic and biotic stresses (Li et al., 2018; Coskun et al., 2019). Although it has not been reported that Si protects potato against the oomycete pathogen *P. infestans*, we observed that a farmer had controlled late blight on potato with the foliar spraying of fungicides containing sodium silicate (Na_2_SiO_3_). It was hypothesized that Si may act as a defense inducer to enhance LB resistance in potato as well as other plants. To determine the Si-mediated LB resistance, a series of concentrations of Na_2_SiO_3_ solution was presprayed on living potato plants 1 day before inoculation with *P. infestans* EC1 using a pot experiment assay. As shown in Figure 1A and Supplementary Figure 1, 100 mM is the best concentration of Na_2_SiO_3_ solution (nominated as Si, hereafter) for protecting the susceptible variety Désirée against EC1. In addition, mycelia growth was not affected on Rye A medium containing 100 mM Na_2_SiO_3_ solution (Supplementary Figure 2). Therefore, 100 mM Si was selected for further investigations.

Then, we conducted the detached leaves assay following inoculation of EC1 on Désirée. As shown in Figure 1B, the disease symptom caused by EC1 was alleviated by foliar pre-spraying of Si. According to statistically analysis, the levels (0–4) of infected leaves, compared to the control of 55.21 and 54.17%, the disease index of Si-pretreated plants are reduced to 35.42 and 18.75% for the living plant assay and detached leaves assays, respectively (Figure 1C). We also examined the local cell death by staining with trypan blue (Weigel and Glazebrook, 2002). During the early infection stage, Si-pretreated leaves showed less staining than the control leaves beginning 2 days post-inoculation (dpi), indicating that the Si-pretreated leaves are more resistant to EC1 (Figure 1D). Overall, these results suggest that foliar application of Si enhances resistance to the oomycete pathogen *P. infestans* in potato plants.

### Si Induces the Accumulation of Superoxide and Hydrogen Peroxide

Si has been observed to induce plant resistance to fungi and bacteria. In this study, we detected the superoxide (O_2_.^–^) and hydrogen peroxide (H_2_O_2_) production in potato leaves using NBT or DAB staining. Compared to the control plant leaves, the Si-treated plant leaves without inoculation had no remarkable difference from 3 to 12 h, whereas the Si-treated plant leaves accumulated more superoxide and hydrogen peroxide at 1 day (0 dpi), upon completion of inoculation with EC1. The significant difference between the Si and control treatments was clear at 1 dpi (Supplementary Figure 3). These results indicate that Si treatment resulted in accumulation of superoxide and hydrogen peroxide in potato leaves during *P. infestans* infection.

### Si Activates the ET and JA Signaling Pathways and Inhibits the SA Signaling Pathway

**FIGURE 2:**
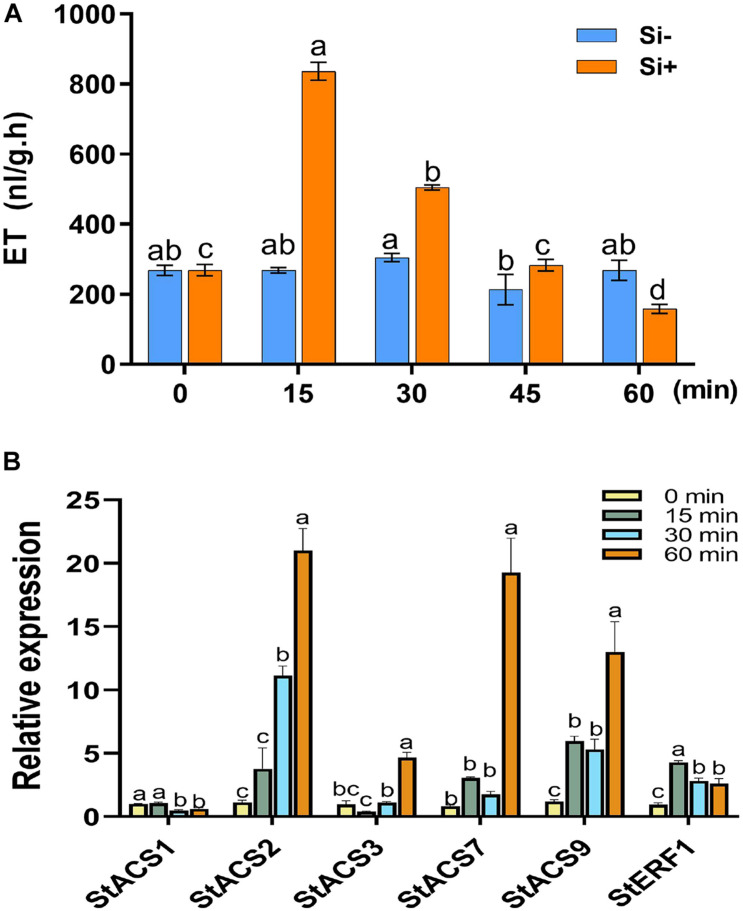
Si activates the transcription of *ACS* genes to promote early ET production in potato. **(A)** Gas chromatography was used to assess the accumulation of ethylene. Each treatment was performed for 5 leaves from different plants. Values are mean ± SE (*n* = 5). Water supplemented with 0.05% (V/V) Tween 20 is used as the control. **(B)** The qRT-PCR for measuring the relative expression of ET synthesis-related genes of *StACS1*, *StACS2*, *StACS3*, *StACS7*, and *StACS9*, and ET-responsive gene *StERF1*. Data are mean ± SE (*n* = 3). An internal marker of *StEF1* gene and the untreated controls (Si–) for each timepoint were used to normalize expression levels. Letters above bars indicate significant difference among treatments (Tukey’s multiple range test, *p* < 0.05). Three biological repeats were performed with similar results.

**FIGURE 3:**
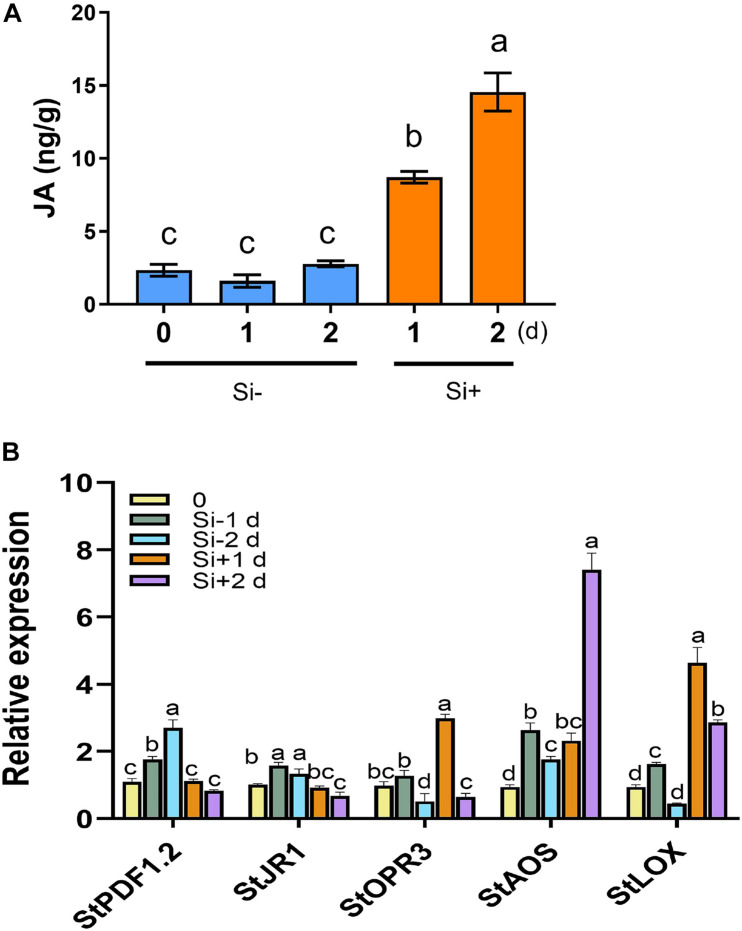
*Phytophthora infestans* inoculation activates JA synthesis following Si treatment. **(A)** JA contents were increased in potato leaves after inoculation with *P. infestans* EC1 following 100 mM Si treatment. Water supplemented with 0.05% (V/V) Tween 20 is used as the control. Three biological repeats were performed with HPLC-MS/MS system. **(B)** The expression patterns of the JA-responsive gene *StPDF1.2* and *StJR1*, the JA synthesis-related genes *StOPR3*, *StAOS*, and *StLOX*. Potato leaves were treated with water or Si, and collected RNA samples at 0, 1, and 2 days (1 day post inoculation with EC1). The *StEF1* gene was used as a control to normalize expression. Above values are mean ± SE (*n* = 3). Letters above bars indicate significant difference among treatments (Tukey’s multiple range test, *p* < 0.05). Three biological repeats were performed with similar results.

**FIGURE 4:**
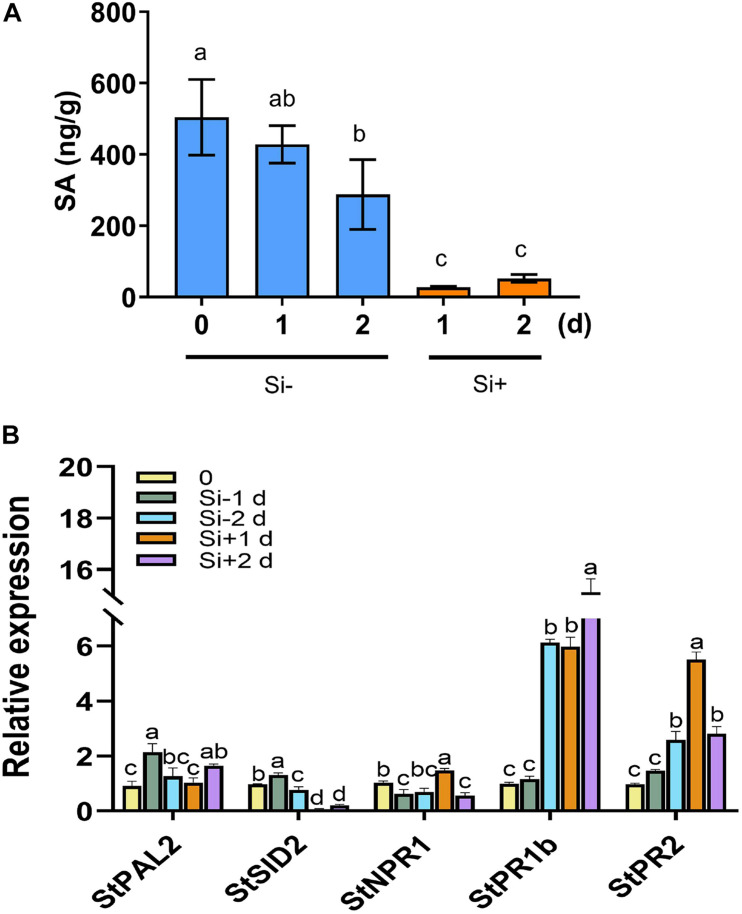
Repressed SA accumulation and activated expression of SA-related *PR* genes after pathogen challenge following Si treatment. **(A)** Levels of SA were quantified by HPLC-MS/MS in potato leaves. Water plus 0.05% (V/V) Tween 20 is used as control. Inoculation with *P. infestans* EC1 at 1 day following 100 mM Si treatment. **(B)** Si treatment repressed the expression of SA synthesis-related genes *StPAL2* and *StSID2*, and activated the expression of SA-induced genes *StNPR1, StPR1*, and *StPR2*. Potato leaves were treated with water or Si, and collected RNA samples at 0, 1, and 2 day (1 day post inoculation with EC1). The *StEF1* gene was used as a control to normalize expression. Letters above bars indicate significant difference among treatments (Tukey’s multiple range test, *p* < 0.05). Three biological repeats were performed with similar results.

The plant hormones ET, JA and SA are involved in potato LB resistance (Liu et al., 2020). To determine the altered hormones levels after foliar treatment of Si, we quantified the hormone levels and the expression of hormone-related genes. As shown in Figure 2A, Si rapidly activates potato leaves to produce ET 15 min after spraying Si without pathogen inoculation. Compared to the control, an approximately threefold increase in ET has been observed at the peak of 15 min after spraying Si; subsequently, it falls to a similar level. Consistent with the rapid accumulation of ET, several ET synthesis-related genes, including *StACS2*, *StACS3*, *StACS7*, and *StACS9* (Peng et al., 2005), and a typical ET-responsive gene of *StERF1* (Solano et al., 1998), were identified to dramatically increase expression at the early stage of Si treatment after normalization to each untreated control (Figure 2B). Foliar treatment of Si also significantly increased the level of JA in potato plants (Figure 3A). Compared to the control, high accumulation of JA was observed at 1 day (0 dpi) and 2 days (1 dpi) after spraying Si. Consistently, Foliar treatment of Si resulted in upregulated expression of several JA biosynthesis-related genes, including *StLOX*, *StAOS*, and *StOPR3* (Figure 3B) (Sasaki et al., 2001; Stenzel et al., 2003; Wasternack, 2007). Surprisingly, the expression of the JA-responsive genes *StPDF1.2* and *StJR1* (Penninckx et al., 1998) was not clearly affected by foliar treatment of Si at 1 day and 1 dpi (Figure 3B). As JA is often negatively correlated with SA, we also observed that the SA level was decreased at 1 day and 1 dpi after Si treatment compared to the control (Figure 4A). Consistent with the decreased content of SA, foliar treatment of Si repressed the expression of the SA biosynthesis-related gene *StSID2* at 1 day (Figure 4B). Si did not significantly induce the expression of the other SA biosynthesis-related gene *StPAL2* (Figure 4B). However, we observed that the expression of the SA-responsive genes *StPR1b* and *StPR2* (Niki et al., 1998) was upregulated at 1 day and restored to normal at 2 days without pathogen inoculation (Supplementary Figure 4), while upregulated at 1 dpi (Si+2d) with EC1 challenged (Figure 4B). Thus, foliar treatment of Si increases the accumulation of ET and JA, which might be the main cause of Si-mediated LB resistance.

### Exogenous Application of MeJA or a JA Biosynthesis Inhibitor Alters Potato Late Blight Resistance

**FIGURE 5:**
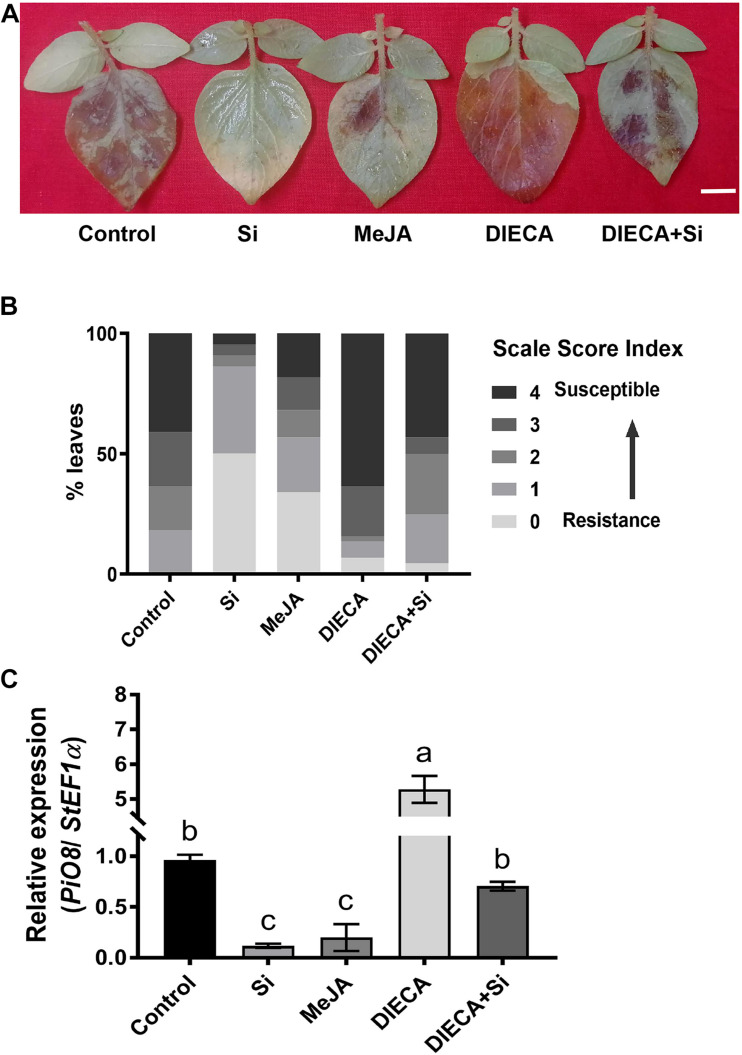
Jasmonic acid positively regulates potato defense to *P. infestans.* **(A)** Disease symptoms of potato leaves inoculated with *P. infestans* following treatment with Si, MeJA, the JA inhibitor DIECA, the mixture of Si and DIECA or control. Pathogen inoculation was performed 1 day after treatment. The image was photographed at 5 dpi. Scale bar represents 1 cm. **(B)** The disease index analysis for **(A)**. Rating scale score of disease index for more than 20 leaves from each of three independent biological replicates. **(C)** The biomass level of *P. infestans* EC1 in potato leaves at 5 dpi for **(A)**. The level of *PiO8* element and *StEF1* were used to quantify the genomic DNA level of EC1 and plant cells by qPCR, respectively. The values are mean ± SE (*n* = 3). Letters above bars indicate significant difference among treatments (Tukey’s multiple range test, *p* < 0.05). Three biological repeats were performed with similar results.

We observed that both ET and JA were accumulated after foliar treatment of Si correlated with enhanced potato LB resistance. Previously, we have identified that ET positively regulates potato LB resistance (Liu et al., 2020). To determine the function of JA on potato LB resistance, we inoculated the potato plants with *P. infestans* EC1 following treatment with Si, MeJA, the JA biosynthesis inhibitor DIECA (Farmer et al., 1994) or the combination of DIECA and Si. The EC1-mediated disease symptom of potato leaves was alleviated after treatment with MeJA or Si compared with the control treatment (Figure 5A). In contrast, potato leaves sprayed with DIECA were more susceptible to EC1 than the control leaves (Figure 5A). In addition, potato leaves treated with the mixture of DIECA and Si showed an intermediate resistance phenotype: greater resistance than the control plants, but greater susceptibility than the Si-treated plants (Figure 5A). Consistently, the disease index (Figure 5B) and the biomass of EC1 in the potato leaves (Figure 5C) supported the altered disease symptom described above. Overall, these results suggest that JA positively regulates potato LB resistance and is required for Si-mediated resistance.

### Si-Mediated Potato Late Blight Resistance Is Dependent on JA/ET and *NPR1*

**FIGURE 6:**
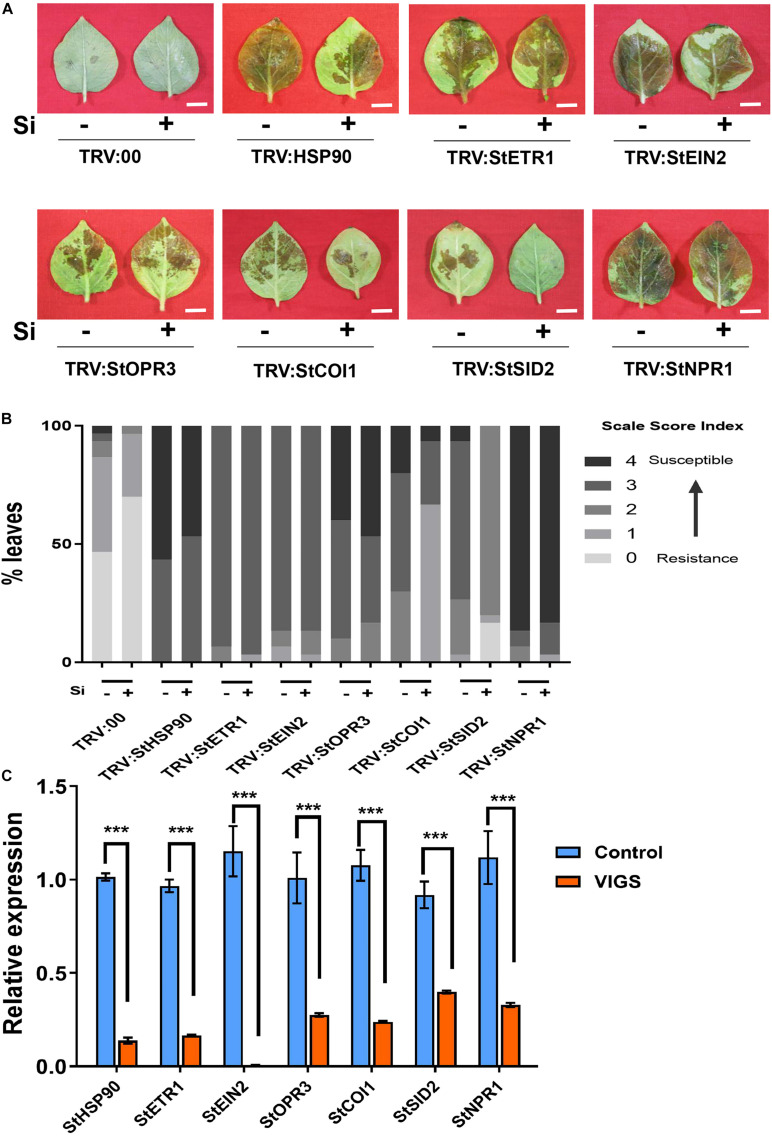
Effect of Si on resistance against *P. infestans* in virus-induced gene silencing plants. Disease symptom **(A)** and disease index **(B)** in response to Si treatment for *StHSP90*-, *StETR1*-, *StEIN2*-, *StOPR3*-, *StCOI1*-, *StSID2*-, and *StNPR1*-VIGS plants. Fifteen leaves from at least three plants were inoculated with *P. infestans* EC1 at 24 h following Si treatment. The images and data were photographed and analyzed at 2.5 dpi, respectively. Scale bar represents 1 cm. **(C)** The transcription level of target genes was quantified by using qRT-PCR in VIGS plants and wild type plants. The *StEF1* gene was used as a control to normalize expression levels. ***Indicates significant differences from control at *P* < 0.001. Triple biological repeats were performed with similar results for above experiments.

The hormones ET, JA, and SA were altered in Si-treated potato leaves that are associated with Si-mediated LB resistance. To determine the role of hormone signaling in Si-mediated LB resistance, a series of VIGS experiments was performed for JA, SA, and ET biosynthesis- or signaling-related genes, including *StOPR3*, *StCOI1*, *StETR1*, *StEIN2, StSID2*, and *StNPR1* (Figure 6A) (Cao et al., 1997; Alonso et al., 1999; Wildermuth et al., 2001). Compared to the empty TRV2 control, all of the above VIGS lines and the negative control *TRV2-StHSP90* were more susceptible to EC1 without foliar treatment of Si (Figures 6A,B). The significant reduction in the expression of their native genes ranged from 60.22 to 99.41% (Figure 6C). Compared to that in the untreated plants, Si-mediated LB resistance was completely compromised in the *StHSP90*-, *StOPR3*-, *StETR1*-, *StEIN2-*, and *StNPR1-*VIGS plants, but it was still present in the *StCOI1-* and *StSID2-*VIGS plants (Figures 6A,B). These results suggest that Si-mediated LB resistance genetically requires *StOPR3*, *StETR1*, *StEIN2*, and *StNPR1* but not *StCOI1* and *StSID2.* As JA is significantly increased in Si-treated potato leaves and plays a positive role in LB resistance, we further silenced another JA synthesis-related gene, *StAOS.* As shown in Supplementary Figure 5, compared to the control plants, the *StAOS-*silenced plants were more susceptible to EC1 and showed attenuated Si-mediated LB resistance similarly to the *StOPR3*-silenced plants described above (Figure 6A).

### Si-Induced Accumulation of JA Is Dependent on the ET Signaling Pathway

**FIGURE 7:**
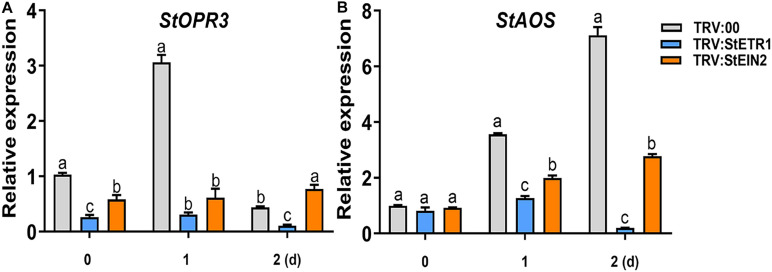
The attenuated induction of the StOPR3 **(A)** and StAOS **(B)** expression in StETR1- andStEIN2-VIGS plants after Si treatment. More than five VIGS plants were treated with Si, and qRT-PCR was performed at 0, 1, and 2 days (1 day post inoculation with *P. infestans* EC1). The *StEF1* gene was used as a control to normalize expression. Letters above bars indicate significant difference among treatments (Tukey’s multiple range test, *p* < 0.05). Three biological repeats were performed with similar results.

**FIGURE 8:**
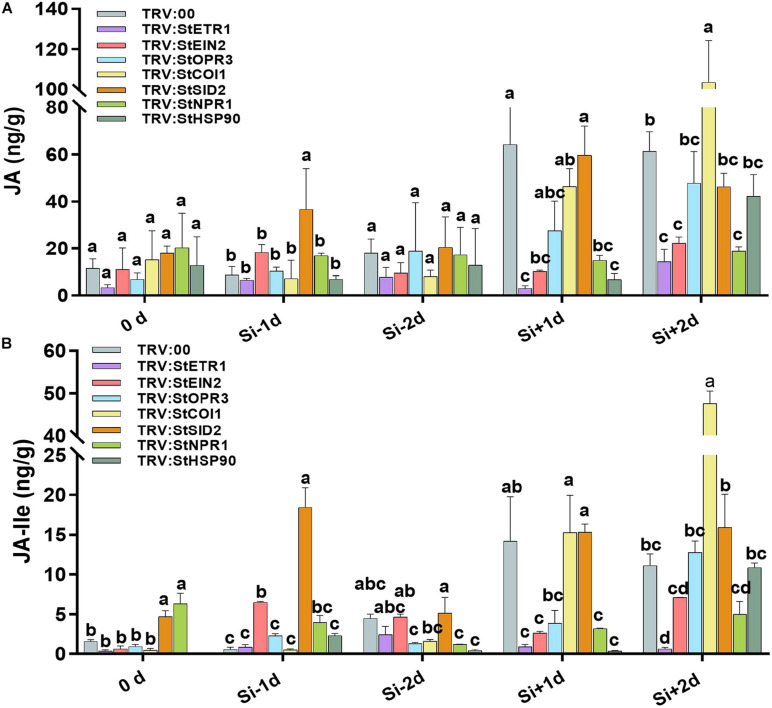
Effect of Si on the contents of JA and JA-Ile in the potato leaves from series of VIGS plants. The levels of JA **(A)** and JA-Ile **(B)** in potato leaves from *StETR1*-, *StEIN2*-, *StOPR3*-, *StCOI1*-, *StSID2*-, *StNPR1*-, and *StHSP90*-VIGS plants or control plants. The samples were collected from non-treatment (0 day), 1 day after water treatment (Si–1d), 1 day post inoculation (dpi) with EC1 following water treatment (Si–2d), 1 day after Si treatment without inoculation (Si+1d), 1 day post-inoculation with EC1 following Si treatment (Si+2d). Values are means ± SE (*n* = 3) from triple independent biological replicates.

Among the phytohormones, JA and ET present a fascinating case of synergism and antagonism. They are commonly recognized as synergistic defense hormones. We have found that both JA and ET participate in defense against *P. infestans*. To explore the cooperation of ET and JA in Si-mediated LB resistance, we noticed that the Si-induced accumulation of ET is faster than JA (Figures 2, 3). Then, we identified that in *StETR1-* and *StEIN2-*VIGS plants, the transcription of *StOPR3* was reduced 8.96- and 3.98-fold, respectively, at 1 day after foliar treatment of Si compared to the control (Figure 7A). Similarly, the transcription of *StAOS* was reduced 1.81- and 0.79-fold of that in control plants, respectively (Figure 7B). Consistent with the expression of JA-related genes, the JA and JA-Ile contents in *StETR1-* and *StEIN2-*VIGS plants was lower than that of the control plants at all five time points (Figures 8A,B). These results indicated that the ET signaling pathway is upstream of JA accumulation in Si-mediated LB resistance.

### The Si-Induced, but Not Pathogen-Induced, Accumulation of JA Is Crucial for Enhanced LB Resistance

The VIGS experiments demonstrated that Si-mediated LB resistance requires *StHSP90*, *StOPR3*, and *StNPR1* but not *StCOI1* and *StSID2.* We measured the JA and JA-Ile contents in VIGS plants and control plants. In control plants, JA and JA-Ile accumulation was identified in Si-treatment 1 day (Si+1d), and pathogen challenge 1 day following foliar treatment of Si 1 day (Si+2d) (Figures 8A,B). In *StHSP90*-, *StOPR3*-, and *StNPR1-*VIGS plants, the significant reduction in JA and JA-Ile accumulation were only identified at Si-treatment 1 day (Si+1d). They still accumulated high contents of JA and JA-Ile at pathogen challenged 1 day (Si+2d) following Si treatment (Figures 8A,B). However, in *StCOI1-* and *StSID2*-VIGS plants, high level accumulations of JA and JA-Ile have been identified the same as the control plants at Si-treatment 1 day (Si+1d) and pathogen challenge 1 day following Si treatment (Si+2d). Particularly, the *StCOI1*-VIGS plants stored a higher level of JA and Ile-JA than the control plants at Si+2d. Given the disease-resistance phenotype of the above VIGS plants after foliar treatment of Si, we concluded that Si-induced accumulation of JA is crucial for enhancing potato LB resistance.

## Discussion

Together with rice and wheat, the potato is an important crop in terms of food security considering the growing population and increasing hunger (Zhang et al., 2017). LB is one of the most devastating potato diseases worldwide and results in an approximately 16% yield loss annually (Kamoun et al., 2015). Si has been broadly used in enhancing plant resistance to abiotic stresses and biotic stresses, particularly in gramineous plants such as rice, maize and sugarcane (Wang et al., 2017; Li et al., 2018; Jang et al., 2018; Parveen et al., 2019). The Si supply has been reported to reduce stalk lodging and to increase tuber dry weight and yield of potato, especially in the absence of water deficiency (Crusciol et al., 2009). Here, we identified that foliar treatment of Si enhanced potato LB resistance, and Si application serves as an extensive strategy to control diseases caused by oomycete pathogens aside from *P. sojae* (Guérin et al., 2014). Notably, in most cases, Si was used for fertilization, where fertilizer uptake by roots expressing Si transporters primes the plant defense responses via SAR (Wang et al., 2017; Li et al., 2018). Previous studies had demonstrated that the expression of *StLsi1* increased in potato roots and leaves upon Si fertilization (Vulavala et al., 2016). Here, we found that foliar treatment of Si enhances potato plant resistance to *P. infestans* by activating several defense responses. Our results also extend the Si application methods beyond Si fertilization of roots.

The production of ethylene is tightly regulated by internal signals during development and in response to environmental stimuli from biotic (e.g., pathogen attack) and abiotic stresses, such as wounding, hypoxia, ozone, chilling, or freezing (Wang et al., 2002). The early production of ET could be found in *Arabidopsis* within 30 min after treatment with MAMPs of flg22 and efl18 (Zipfel et al., 2006) and heavy metal ions of copper and cadmium (Schellingen et al., 2014; Zhang et al., 2018). We also found that Cu^2+^ activates the expression of *StACS* genes and ET production in a very short time period (15–30 min) in potato (Liu et al., 2020). Here, we found that the expression of several *StACS* genes and ET production were activated by Si within 15 min without pathogen challenge in potato (Figures 2A,B), suggesting that Si might act as an elicitor like Cu^2+^ or MAMPs. The *StETR1-* and *StEIN2-*VIGS plants showed a more susceptible phenotype than wild type. They also failed in the Si-mediated defense response to *P. infestans* (Figure 6A). Based on the attenuated expression of JA-related genes and the content of JA and JA-Ile in *StETR1-* and *StEIN2-*VIGS plants (Figures 7, 8), we concluded that rapidly induced ET synthesis is required for Si-mediated LB resistance in potato.

Several metabolites of jasmonates (JA) have been reported to act as signaling molecules in triggering plant immunity. Among them, (+)-7-iso-jasmonoyl- L -isoleucine (JA-Ile) is the major bioactive form of the hormone JA (Kombrink, 2012; Wasternack and Hause, 2013). JA-Ile may be a mobile signal involved in the induction of ISR, which is triggered mostly by biocontrol agents and necrotrophic fungi (Kravchuk et al., 2011). Kim et al. (2014) reported that JA levels were significantly increased in Si-treated rice plants under normal conditions. A similar finding was acquired from Si treated potato in this study (Figure 5). We found that exogenous MeJA application alone increased resistance to *P. infestans* EC1. Contrarily, the Si-mediated resistance was inhibited by DIECA (Figure 5). The *StAOS*-, *StLOX-*, and *StOPR3-*VIGS plants showed significant reductions in JA and JA-Ile content and alleviated resistance to EC1 with Si treatment (Figure 6). Thus, our result demonstrated that foliar application of Si enhances the resistance to LB dependent on increased JA contents. In rice, both JA accumulation and signaling pathway are required for Si-mediated resistance to insect herbivory (Ye et al., 2013). However, we found that repressing the expression of *StCOI1* does not affect the high accumulation of JA and Si-mediated LB resistance in potato (Figures 6, 8). This is consistent with recent findings in *Arabidopsis*. Wang et al. (2020) found that Si-treatment will enhance the accumulation of JA and the resistance to powdery mildew. However, Si fertilization still enhances powdery mildew in the *Arabidopsis coi1* mutant (Wang et al., 2020). Overall, we conclude that JA biosynthesis, but not JA signaling pathway, is required to induce Si-mediated LB resistance. In *StETR1-* and *StEIN2-*VIGS plants, JA accumulation and Si-induced transcription of *StOPR3*, *StLOX*, and *StAOS* are inhibited (Figures 7, 8). These results indicated that JA-related Si-mediated LB resistance is downstream of the ET signaling pathway.

Salicylic acid, an antagonistic phytohormone to JA, plays an important role in plant immunity, including resistance in both local and systemic tissue upon biotic attack, hypersensitive responses, and cell death (Ding and Ding, 2020). In this study, we have found that they shared common roles in Si-mediated LB resistance. Foliar spraying of Si repressed SA accumulation while activating the accumulation of JA and JA-Ile on potato plants (Figures 4, 5). Surprisingly, Si-mediated LB resistance was still present in the *StSID2-*VIGS potato plants (Figure 6), suggesting that it is independent of SA synthesis. Previously, heterozygous expression of wheat Si transporter in the *Arabidopsis sid2* or *pad4* background have also revealed a significantly reduced SA content and more resistance to *G. cichoracearum* than control plants following Si treatment (Vivancos et al., 2015). *NPR1* is a master regulator of the SA-mediated induction of defense genes downstream of *SID2*. It is positively regulated by several SA-inducible WRKY proteins (Cao et al., 1997; Yu et al., 2001). Accumulating evidence points to an unknown cytosolic function of *NPR1* in the JA/ET signaling pathway and ISR (Pieterse et al., 2002; Zhou and Wang, 2018). In this study, Si-mediated resistance against *P. infestans* required *NPR1* and was completely attenuated in the *StNPR1-*VIGS plants (Figure 6), suggesting a signaling pathway independent of SA but dependent on NPR1 for Si-mediated LB resistance. This activity is reminiscent of the ISRs triggered by the rhizobacterium *P. fluorescens* WCS417r and *B. cereus* AR156 against *Pst* DC3000 and *B. cinerea* in *Arabidopsis* (Pieterse et al., 2002; Nie et al., 2017).

## Conclusion

**FIGURE 9:**
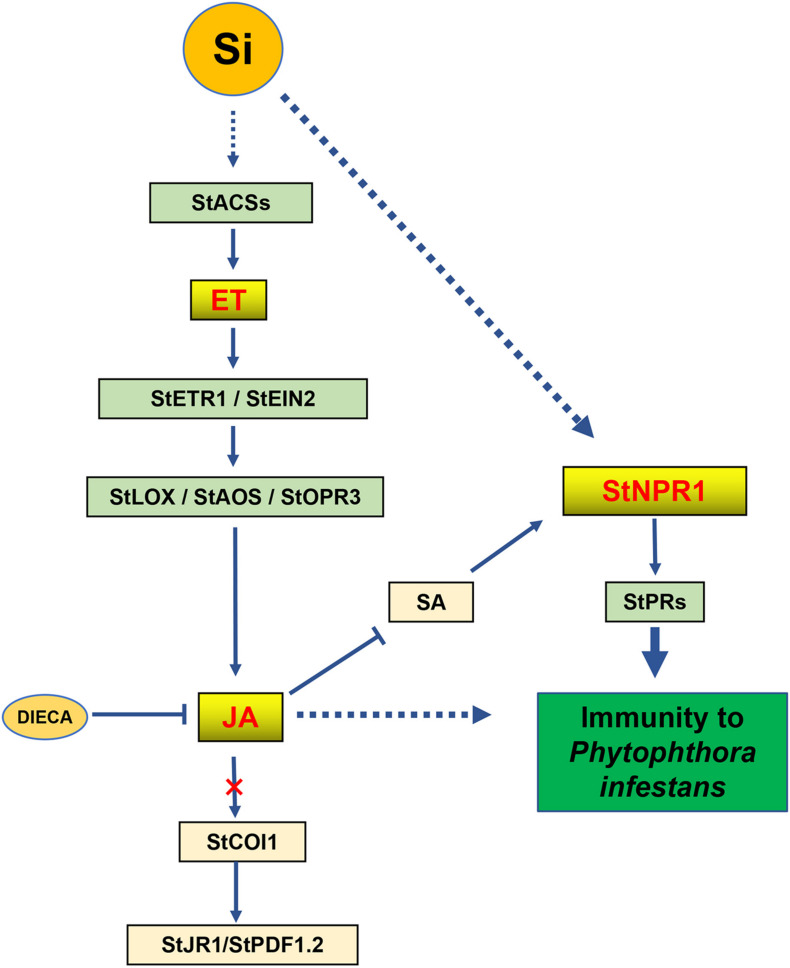
A proposed network of phytohormones for Si-mediated potato late blight resistance. Foliar spraying Si triggers the early ET accumulation in potato leaves. It induces the expression of JA synthesis-related genes to increase the levels of JA and JA-Ile and to suppress the level of SA. However, Si-mediated resistance is required for JA accumulation and independent on JA signaling pathway. Moreover, independent on SA signaling pathway, Si specifically activates StNPR1 induce the expression of *NPR1*-dependent *PR* genes to mediate the resistance against *P. infestans*.

Our results demonstrated that foliar spraying with Si enhances the resistance of potato against *P. infestans*. The ET, JA and SA phytohormone signaling pathways are involved in Si-mediated immunity. Similar to the activity of several biocontrol agents, Si-mediated LB resistance is dependent on ET, JA, and NPR1, as described in Figure 9.

## Data Availability Statement

The original contributions generated for this study are included in the article/Supplementary Material, further inquiries can be directed to the corresponding author/s.

## Author Contributions

ZC and WY conceived and designed the experiments. XX, TG, HL, and WY performed the experiments. XX and CZ analyzed the data. XX, WY, and ZC wrote the manuscript. All authors read and approved the final manuscript.

## Conflict of Interest

The authors declare that the research was conducted in the absence of any commercial or financial relationships that could be construed as a potential conflict of interest.

## Footnotes

1 https://www.graphpad.com

## Supplementary Material

Supplementary Figure 1 Effects of Si on the growth of *P. infestans in vitro*. Scale bar represents 1 cm. **(A)** The growth of *P. infestans* EC1 on the Rye A medium (control) or Rye A containing 100 mM Na_2_SiO_3_. The images were photographed at 7 days. **(B)** The colony diameter of *P. infestans* EC1 in **(A)**. Data represent the mean ± SD (*n* = 18). *ns* indicates no significant differences compared with control.

Supplementary Figure 2 The disease index of potato resistance to *P. infestans* after foliar spraying with 10, 50, 100, 500, or 1,000 mM Si. Over 30 leaves from 10 plants were scored for 0 to 4 levels.

Supplementary Figure 3 Accumulation of ROS in the inoculated potato leaves in response to Si treatment. Potato leaves were stained with DAB and NBT to assess the accumulation of the hydrogen peroxide (H_2_O_2_) and superoxide (O_2_.^–^), respectively. Each three plants were sprayed with Si or control (Water), subsequently inoculated with *P. infestans* EC1 after 24 h. The experiments were repeated three times with similar results. Scale bar represents 1 cm.

Supplementary Figure 4 The relative expression of *StPR1b* and *StPR2* under foliar treatment of Si without EC1 inoculation. The expression of *StPR1b* **(A)** and *StPR2* **(B)** with or without foliar treatment of Si. Potato leaves were treated with water or Si, and collected RNA samples at 0, 1, and 2 days. The *StEF1* gene was used as a control to normalize expression. Letters above bars indicate significant difference among treatments (Tukey’s multiple range test, *p* < 0.05). Three biological repeats were performed with similar results.

Supplementary Figure 5 The Si-mediated resistance was alleviated in *StAOS*-VIGS plants. Disease symptom **(A)** and disease index **(B)** in response to Si treatment. Scale bar represents 1 cm. Fifteen leaves from five individuals were inoculated with *P. infestans* EC1 at 24 h following Si treatment. The images and data were photographed and analyzed at 2.5 dpi, respectively. **(C)** The transcription level of *StAOS* were dramatically repressed in VIGS plants quantified by using qRT-PCR. The *StEF1* gene was used as a control to normalize expression levels. ^∗∗∗^Indicates significant differences from control at *P* < 0.001. Triple biological repeats were performed with similar results.

Supplementary Table 1 Strains and plasmids and other materials used in this study.

Supplementary Table 2 List of oligonucleotide primers used in this study.

## References

[B1] Alonso J. M. Hirayama T. Roman G. Nourizadeh S. Ecker J. R. (1999). EIN2, a bifunctional transducer of ethylene and stress responses in *Arabidopsis*. *Science* 284 2148–2152. 10.1126/science.284.5423.2148 10381874

[B2] Assis F. A. Moraes J. C. Auad A. M. Coelho M. (2013). The effects of foliar spray application of silicon on plant damage levels and components of larval biology of the pest butterfly *Chlosyne lacinia* saundersii (*Nymphalidae*). *Int. J. Pest Manag.* 59 128–134. 10.1080/09670874.2013.779049

[B3] Bae H. Kim M. S. Sicher R. C. Bae H. J. Bailey B. A. (2006). Necrosis- and ethylene-inducing peptide from *Fusarium oxysporum* induces a complex cascade of transcripts associated with signal transduction and cell death in *Arabidopsis*. *Plant Physiol.* 141 1056–1067. 10.1104/pp.106.076869 16698904 PMC1489885

[B4] Brackhage C. Schaller J. Bäucker E. Dudel E. G. (2013). Silicon availability affects the stoichiometry and content of calcium and micro nutrients in the leaves of common reed. *Silicon* 5 199–204. 10.1007/s12633-013-9145-3

[B5] Brigneti G. Martín-Hernández A. M. Jin H. Chen J. Baulcombe D. C. Baker B. (2004). Virus-induced gene silencing in *Solanum species*. *Plant J.* 39 264–272. 10.1111/j.1365-313X.2004.02122.x 15225290

[B6] Cao H. Glazebrook J. Clarke J. D. Volko S. Dong X. (1997). The *Arabidopsis* NPR1 gene that controls systemic acquired resistance encodes a novel protein containing ankyrin repeats. *Cell* 88 57–63. 10.1016/s0092-8674(00)81858-9 9019406

[B7] Caten C. E. Jinks J. L. (1968). Spontaneous variability of single isolates of *Phytophthora infestans*. *I. Cultural variation. Can. J. Bot.* 48 897–905. 10.1139/b68-055

[B8] Coskun D. Britto D. T. Huynh W. Q. Kronzucker H. J. (2016). The role of silicon in higher plants under salinity and drought stress. *Front. Plant Sci.* 7:1072. 10.3389/fpls.2016.01072 27486474 PMC4947951

[B9] Coskun D. Deshmukh R. Sonah H. Menzies J. G. Reynolds O. Ma J. F. (2019). The controversies of silicon’s role in plant biology. *New Phytol.* 221 67–85. 10.1111/nph.15343 30007071

[B10] Crusciol C. A. C. Pulz A. L. Lemos L. B. Soratto R. P. Lima G. P. P. (2009). Effects of silicon and drought stress on tuber yield and leaf biochemical characteristics in potato. *Crop Sci.* 49 949–954. 10.2135/cropsci2008.04.0233

[B11] Datnoff L. E. Elmer W. H. Huber D. M. (2007). *Mineral Nutrition and Plant Disease.* St. Paul, MN: The American Phytopathological Society.

[B12] Denoux C. Galletti R. Mammarella N. Gopalan S. Werck D. De Lorenzo G. (2008). Activation of defense response pathways by OGs and Flg22 elicitors in *Arabidopsis* seedlings. *Mol. Plant.* 1 423–445. 10.1093/mp/ssn019 19825551 PMC2954645

[B13] Ding P. Ding Y. (2020). Stories of salicylic acid: a plant defense hormone. *Trends Plant Sci.* 25 549–565. 10.1016/j.tplants.2020.01.004 32407695

[B14] Doares S. H. Narvaez-Vasquez J. Conconi A. Ryan C. A. (1995). Salicylic acid inhibits synthesis of proteinase inhibitors in tomato leaves induced by systemin and Jasmonic acid. *Plant Physiol.* 108 1741–1746. 10.1104/pp.108.4.1741 12228577 PMC157556

[B15] Durrant W. E. Dong X. (2004). Systemic acquired resistance. *Annu. Rev. Phytopathol.* 42 185–209. 10.1146/annurev.phyto.42.040803.140421 15283665

[B16] Farmer E. E. Caldelari D. Pearce C. Walker-Simmons M. K. Ryan C. A. (1994). Diethyldithiocarbamic acid inhibits the octadecanoid signaling pathway for the wound induction of proteinase inhibitors in tomato leaves. *Plant Physiol.* 106 337–342. 10.1104/pp.106.1.337

[B17] Ghareeb H. Bozsó Z. Ott P. G. Repenning C. Stahl F. Wydra K. (2011). Transcriptome of silicon-induced resistance against *Ralstonia solanacearum*, in the silicon non-accumulator tomato implicates priming effect. *Physiol. Mol. Plant Pathol.* 75 83–89. 10.1007/s11104-014-2293-4

[B18] Guérin V. Lebreton A. Cogliati E. E. Hartley S. E. Belzile F. Menzies J. G. (2014). A zoospore inoculation method with *Phytophthora sojae* to assess the prophylactic role of silicon on soybean cultivars. *Plant Dis.* 98 1632–1638. 10.1094/pdis-01-14-0102-re 30703877

[B19] Heine G. Tikum G. Horst W. J. (2006). The effect of silicon on the infection by and spread of *Pythium aphanidermatum* in single roots of tomato and bitter gourd. *J. Exp. Bot.* 58 569–577. 10.1093/jxb/erl232 17158106

[B20] Hodson M. J. White P. J. Mead A. Broadley M. R. (2005). Phylogenetic variation in the silicon composition of plants. *Ann. Bot.* 96 1027–1046. 10.1093/aob/mci255 16176944 PMC4247092

[B21] Jang S. W. Kim Y. Khan A. L. Na C. I. Lee I. J. (2018). Exogenous short-term silicon application regulates macro-nutrients, endogenous phytohormones, and protein expression in *Oryza sativa* L. *BMC Plant Biol.* 18:4. 10.1186/s12870-017-1216-y 29301510 PMC5755014

[B22] Jiang N. Fan X. Lin W. Wang G. Cai K. (2019). Transcriptome analysis reveals new insights into the bacterial wilt resistance mechanism mediated by silicon in tomato. *Int. J. Mol. Sci.* 20:761. 10.3390/ijms20030761 30754671 PMC6387441

[B23] Judelson H. S. Tooley P. W. (2000). Enhanced polymerase chain reaction methods for detecting and quantifying *Phytophthora infestans* in plants. *Phytopathology* 90 1112–1119. 10.1094/PHYTO.2000.90.10.1112 18944474

[B24] Kamoun S. Furzer O. Jones J. D. Judelson H. S. Ali G. S. Dalio R. J. (2015). The top 10 oomycete pathogens in molecular plant pathology. *Mol. Plant Pathol.* 16 413–434. 10.1111/mpp.12190 25178392 PMC6638381

[B25] Kim S. G. Kim K. W. Park E. W. Choi D. (2002). Silicon-induced cell wall fortification of rice leaves: a possible cellular mechanism of enhanced host resistance to blast. *Phytopathology* 92 1095–1103. 18944220 10.1094/PHYTO.2002.92.10.1095

[B26] Kim Y. H. Khan A. L. Kim D. H. Lee S. Y. Kim K. M. Waqas M. (2014). Silicon mitigates heavy metal stress by regulating P-type heavy metal ATPases, *Oryza sativa* low silicon genes, and endogenous phytohormones. *BMC Plant Biol.* 14:13. 10.1186/1471-2229-14-13 24405887 PMC3893592

[B27] Klarzynski O. Plesse B. Joubert J. M. Yvin J. C. Kopp M. Kloareg B. (2000). Linear beta-1,3 glucans are elicitors of defense responses in tobacco. *Plant Physiol.* 124 1027–1038. 10.1104/pp.124.3.1027 11080280 PMC59202

[B28] Kombrink E. (2012). Chemical and genetic exploration of jasmonate biosynthesis and signaling paths. *Planta* 236 1351–1366. 10.1007/s00425-012-1705-z 23011567

[B29] Kravchuk Z. Vicedo B. Flors V. Camańes G. González-Bosch C. Garcia-Agustin P. (2011). Priming for JA-dependent defenses using hexanoic acid is an effective mechanism to protect *Arabidopsis* against *B. cinerea*. *J. Plant Physiol.* 186 359–366. 10.1016/j.jplph.2010.07.028 20950893

[B30] Kvedaras O. L. An M. Choi Y. S. Gurr G. M. (2010). Silicon enhances natural enemy attraction and biological control through induced plant defences. *Bull. Entomol. Res.* 100 367–371. 10.1017/S0007485309990265 19737442

[B31] Lee S. K. Sohn E. Y. Hamayun M. Yoon J. Y. Lee I. J. (2010). Effect of silicon on growth and salinity stress of soybean plant grown under hydroponic system. *Agrofor. Syst.* 80 333–340. 10.1007/s10457-010-9299-6

[B32] Lehtonen M. T. Akita M. Frank W. Reski R. Valkonen J. P. (2012). Involvement of a class III peroxidase and the mitochondrial protein TSPO in oxidative burst upon treatment of moss plants with a fungal elicitor. *Mol. Plant Microbe Interact.* 25 363–371. 10.1094/MPMI-10-11-0265 22112216

[B33] Li Z. Song Z. Yan Z. Hao Q. Song A. Liu L. (2018). Silicon enhancement of estimated plant biomass carbon accumulation under abiotic and biotic stresses. A meta-analysis. *Agron. Sustain. Dev.* 38:26 10.1007/s13593-018-0496-4

[B34] Lin Y. Sun Z. Li Z. Xue R. Cui W. Sun S. (2019). Deficiency in silicon transporter Lsi1 compromises inducibility of anti-herbivore defense in rice plants. *Front. Plant Sci.* 10:652. 10.3389/fpls.2019.00652 31178878 PMC6543919

[B35] Liu H. Xue X. Yu Y. Xu M. Lu C. Meng X. (2020). Copper ions suppress abscisic acid biosynthesis to enhance defense against *Phytophthora infestans* in potato. *Mol. Plant Pathol.* 21 636–651. 10.1111/mpp.12919 32077242 PMC7170774

[B36] Liu H. Zhang B. Wu T. Ding Y. Ding X. Chu Z. (2015). Copper ion elicits defense response in *Arabidopsis thaliana* by activating salicylate- and ethylene-dependent signaling pathways. *Mol. Plant* 8 1550–1553. 10.1016/j.molp.2015.07.008 26225489

[B37] Lund S. T. Stall R. E. Klee H. J. (1998). Ethylene regulates the susceptible response to pathogen infection in tomato. *Plant Cell* 10 371–382. 10.1105/tpc.10.3.371 9501111 PMC144005

[B38] Malamy J. Carr J. P. Klessig D. F. Raskin I. (1990). Salicylic acid: a likely endogenous signal in the resistance response of tobacco to viral infection. *Science* 250 1002–1004. 10.1126/science.250.4983.1002 17746925

[B39] Muneer S. Park Y. G. Kim S. Jeong B. R. (2017). Foliar or subirrigation silicon supply mitigates high temperature stress in strawberry by maintaining photosynthetic and stress-responsive proteins. *J. Plant Growth Regul.* 36 836–845. 10.1007/s00344-017-9687-5

[B40] Nascimento K. J. T. Debona D. França S. K. S. Gonçalves M. G. M. DaMatta F. M. Rodrigues F. Á (2014). Soybean resistance to *Cercospora sojina* infection is reduced by silicon. *Phytopathology* 104 1183–1191. 10.1094/PHYTO-02-14-0047-R 24805073

[B41] Nie P. Li X. Wang S. Guo J. Zhao H. Niu D. (2017). Induced systemic resistance against *Botrytis cinerea* by *Bacillus cereus* AR156 through a JA/ET- and NPR1-dependent signaling pathway and activates PAMP-triggered immunity in *Arabidopsis*. *Front. Plant Sci.* 8:238. 10.3389/fpls.2017.00238 28293243 PMC5329000

[B42] Niki T. Mitsuhara I. Seo S. Ohtsubo N. Ohasshi Y. (1998). Antagonistic effect of salicylic acid and jasmonic acid on the expression of pathogenesis-related (PR) protein genes in wounded mature tobacco leaves. *Plant Cell Physiol.* 39 500–507. 10.1093/oxfordjournals.pcp.a029397

[B43] Parveen A. Liu W. Hussain S. Asghar J. Perveen S. Xiong Y. (2019). Silicon priming regulates morpho-physiological growth and oxidative metabolism in maize under drought stress. *Plants.* 8:431. 10.3390/plants8100431 31635179 PMC6843370

[B44] Peng H. P. Lin T. Y. Wang N. N. Shih M. C. (2005). Differential expression of genes encoding 1-aminocyclopropane-1-carboxylate synthase in *Arabidopsis* during hypoxia. *Plant Mol. Biol.* 58 15–25. 10.1007/s11103-005-3573-4 16028113

[B45] Penninckx I. A. Thomma B. P. Buchala A. Metraux J. P. Broekaert W. F. (1998). Concomitant activation of jasmonate and ethylene response pathways is required for induction of a plant defensin gene in *Arabidopsis*. *Plant Cell* 10 2103–2113. 10.1105/tpc.10.12.2103 9836748 PMC143966

[B46] Pieterse C. M. J. Van Wees S. C. M. Ton J. Van Pelt J. A. Van Loon L. C. (2002). Signalling in rhizobacteria-induced systemic resistance in *Arabidopsis thaliana*. *Plant Biol.* 4 535–544. 10.1055/s-2002-35441

[B47] Qiu A. Liu Z. Li J. Chen Y. Guan D. He S. (2016). The ectopic expression of CaRop1 modulates the response of tobacco plants to *Ralstonia solanacearum* and aphids. *Front. Plant Sci.* 7:1177. 10.3389/fpls.2016.01177 27551287 PMC4976107

[B48] Rasoolizadeh A. Labbé C. Sonah H. Deshmukh R. K. Belzile F. Menzies J. G. (2018). Silicon protects soybean plants against *Phytophthora sojae* by interfering with effector-receptor expression. *BMC Plant Biol.* 18:97. 10.1186/s12870-018-1312-7 29848307 PMC5977513

[B49] Ratcliff F. Martin-Hernandez A. M. Baulcombe D. C. (2001). Tobacco rattle virus as a vector for analysis of gene function by silencing. *Plant J.* 25 237–245. 10.1046/j.0960-7412.2000.00942.x 11169199

[B50] Rémus-Borel W. Menzies J. G. Belanger R. R. (2005). Silicon induces antifungal compounds in powdery mildew-infected wheat. *Physiol. Mol. Plant Pathol.* 66 108–115. 10.1016/j.pmpp.2005.05.006

[B51] Reynolds O. L. Keeping M. G. Meyer J. H. (2009). Silicon-augmented resistance of plants to herbivorous insects: a review. *Ann. Appl. Biol.* 155 171–186. 10.1111/j.1744-7348.2009.00348.x

[B52] Rodrigues F. A. Duarte H. D. Rezende D. C. Filho J. A. Korndorfer G. H. Zambolim L. (2010). Foliar spray of potassium silicate on the control of angular leaf spot on beans. *Mol. Plant Pathol.* 33 2082–2093. 10.1080/01904167.2010.519082

[B53] Sasaki Y. Asamizu E. Shibata D. Nakamura Y. Kaneko T. Awai K. (2001). Monitoring of methyl jasmonate-responsive genes in *Arabidopsis* by cDNA macroarray: self-activation of jasmonic acid biosynthesis and crosstalk with other phytohormone signaling pathways. *DNA Res.* 8 153–161. 10.1093/dnares/8.4.153 11572481

[B54] Schellingen K. van der Straeten D. Vandenbussche F. Prinsen E. Remans T. Vangronsveld J. (2014). Cadmium-induced ethylene production and response in *Arabidopsis thaliana* rely on ACS2 and ACS6 gene expression. *BMC Plant Biol.* 14:214. 10.1186/s12870-014-0214-6 25082369 PMC4236733

[B55] Shen X. F. Zhou Y. Y. Duan L. S. Li Z. H. Eneji A. E. Li J. M. (2010). Silicon effects on photosynthesis and antioxidant parameters of soybean seedlings under drought and ultraviolet-B radiation. *J. Plant Physiol.* 167 1248–1252. 10.1016/j.jplph.2010.04.011 20713250

[B56] Solano R. Stepanova A. Chao Q. Ecker J. R. (1998). Nuclear events in ethylene signaling: a transcriptional cascade mediated Ethyleneinsensitive3 and ethylene-response-factor1. *Genes & Dev.* 12 3703–3714. 10.1101/gad.12.23.3703 9851977 PMC317251

[B57] Stenzel I. Hause B. Miersch O. Kurz T. Maucher H. Weichert H. (2003). Jasmonate biosynthesis and the allene oxide cyclase family of *Arabidopsis thaliana*. *Plant Mol. Biol.* 51 895–911. 10.1023/a:1023049319723 12777050

[B58] Summermatter K. Sticher L. Metraux J. P. (1995). Systemic responses in *Arabidopsis thaliana* infected and challenged with *Pseudomonas syringae* pv syringae. *Plant Physiol.* 108 1379–1385. 10.1104/pp.108.4.1379 12228548 PMC157515

[B59] Sun W. Zhang J. Fan Q. Xue G. Li Z. Liang Y. (2010). Silicon-enhanced resistance to rice blast is attributed to silicon-mediated defence resistance and its role as physical barrier. *Eur. J. Plant Pathol.* 128 39–49.

[B60] van Bockhaven J. Spíchal L. Novák O. Strnad M. Asano T. Kikuchi S. (2015). Silicon induces resistance to the brown spot fungus *Cochliobolus miyabeanus* by preventing the pathogen from hijacking the rice ethylene pathway. *New Phytol.* 206 761–773. 10.1111/nph.13270 25625327

[B61] Vijayan P. Shockey J. Lévesque C. A. Cook R. J. Browse J. (1998). A role for jasmonate in pathogen defense of *Arabidopsis*. *Proc. Natl. Acad. Sci. U.S.A.* 95 7209–7214. 10.1073/pnas.95.12.7209 9618564 PMC22783

[B62] Vivancos J. Labbé C. Menzies J. G. Bélanger R. R. (2015). Silicon-mediated resistance of *Arabidopsis* against powdery mildew involves mechanisms other than the salicylic acid (SA)-dependent defence pathway. *Mol. Plant Pathol.* 16 572–582. 10.1111/mpp.12213 25346281 PMC6638373

[B63] Vleeshouwers V. G. A. A. van Dooijeweert W. Keizer L. C. P. Sijpkes L. Govers F. Colon L. T. (1999). A laboratory assay for *Phytophthora infestans* resistance in various *Solanum species* reflects the field situation. *Eur. J. Plant Pathol.* 105 241–250. 10.1023/A:1008710700363

[B64] Vulavala V. K. R. Elbaum R. Yermiyahu U. Fogelman E. Kumar A. Ginzberg I. (2016). Silicon fertilization of potato: expression of putative transporters and tuber skin quality. *Planta* 243 217–229. 10.1007/s00425-015-2401-6 26384982

[B65] Wang J. Gao C. Li L. Cao W. Dong R. Ding X. (2019). Transgenic RXLR effector PITG_15718.2 suppresses immunity and reduces vegetative growth in potato. *Int. J. Mol. Sci.* 20:3031. 10.3390/ijms20123031 31234322 PMC6627464

[B66] Wang K. L. C. Li H. Ecker J. R. (2002). Ethylene biosynthesis and signaling networks. *Plant Cell* 14 131–151. 10.1105/tpc.001768 12045274 PMC151252

[B67] Wang L. Dong M. Zhang Q. Wu Y. Hu L. Parson J. F. (2020). Silicon modulates multi-layered defense against powdery mildew in *Arabidopsis*. *bioRxiv [Preprint]* 10.1101/2020.02.05.935734

[B68] Wang M. Gao L. Dong S. Sun Y. Shen Q. Guo S. (2017). Role of silicon on plant-pathogen interactions. *Front. Plant Sci.* 8:701. 10.3389/fpls.2017.00701 28529517 PMC5418358

[B69] Wasternack C. (2007). Jasmonates: an update on biosynthesis, signal transduction and action in plant stress response, growth and development. *Ann. Bot.* 100 681–697. 10.1093/aob/mcm079 17513307 PMC2749622

[B70] Wasternack C. Hause B. (2013). Jasmonates: biosynthesis, perception, signal transduction and action in plant stress response, growth and development. An update to the 2007 review in annals of botany. *Ann. Bot.* 111 1021–1058. 10.1093/aob/mct067 23558912 PMC3662512

[B71] Weigel D. Glazebrook J. (2002). *Arabidopsis: a laboratory manual*, Vol. 80 New York, NY: Cold Spring Harbor Laboratory Press, 77–77. 10.1017/S0016672302215852

[B72] Wildermuth M. C. Dewdney J. Wu G. Ausubel F. M. (2001). Isochorismate synthase is required to synthesize salicylic acid for plant defence. *Nature* 414 562–565. 10.1038/35107108 11734859

[B73] Wohlgemuth H. Mittelstrass K. Kschieschan S. Bender J. Weigelm H. J. Overmyerm K. (2002). Activation of an oxidative burst is a general feature of sensitive plants exposed to the air pollutant ozone. *Plant Cell Environ.* 25 717–726. 10.1046/j.1365-3040.2002.00859.x

[B74] Xu Q. Truong T. T. Barrero J. M. Jacobsen J. V. Hocart C. H. Gubler F. (2016). A role for jasmonates in the release of dormancy by cold stratification in wheat. *J. Exp. Bot.* 67 3497–3508. 10.1093/jxb/erw172 27140440 PMC4892733

[B75] Yang W. Zhang B. Qi G. Shang L. Liu H. Ding X. (2019). Identification of the phytosulfokine receptor 1 (OsPSKR1) confers resistance to bacterial leaf streak in rice. *Planta* 250 1603–1612. 10.1007/s00425-019-03238-8 31388828

[B76] Ye M. Song Y. Long J. Wang R. Baerson S. R. Pan Z. (2013). Priming of jasmonate-mediated antiherbivore defense responses in rice by silicon. *Proc. Natl. Acad. Sci. U.S.A.* 110 E3631–E3639. 10.1073/pnas.1305848110 24003150 PMC3780902

[B77] Yu D. Chen C. Chen Z. (2001). Evidence for an important role of WRKY DNA binding proteins in the regulation of NPR1 gene expression. *Plant Cell* 13 1527–1540. 10.1105/TPC.010115 11449049 PMC139550

[B78] Zhang B. Liu H. Ding X. Qiu J. Zhang M. Chu Z. (2018). *Arabidopsis thaliana* ACS8 plays a crucial role in the early biosynthesis of ethylene elicited by copper ions. *J. Cell Sci.* 131 jcs202424. 10.1242/jcs.202424 28775152

[B79] Zhang H. Xu F. Wu Y. Hu H. H. Dai X. F. (2017). Progress of potato staple food research and industry development in China. *J. Integr. Agric.* 16 2924–2932. 10.1016/S2095-3119(17)61736-2

[B80] Zhou M. Wang W. (2018). Recent advances in synthetic chemical inducers of plant immunity. *Front. Plant Sci.* 9:1613. 10.3389/fpls.2018.01613 30459795 PMC6232518

[B81] Zipfel C. Kunze G. Chinchilla D. Caniard A. Jones J. D. G. Boller T. (2006). Perception of the bacterial MAMP EF-Tu by the receptor EFR restricts *Agrobacterium*-mediated transformation. *Cell* 125 749–760. 10.1016/j.cell.2006.03.037 16713565

