# Supplementary figures and images for: Foliar Application of Silicon Enhances Resistance Against Phytophthora infestans Through the ET/JA- and NPR1- Dependent Signaling Pathways in Potato
Source: Front Plant Sci. 2021 Jan 28;12:609870. doi: 10.3389/fpls.2021.609870 (PMC7876464; doi:10.3389/fpls.2021.609870)

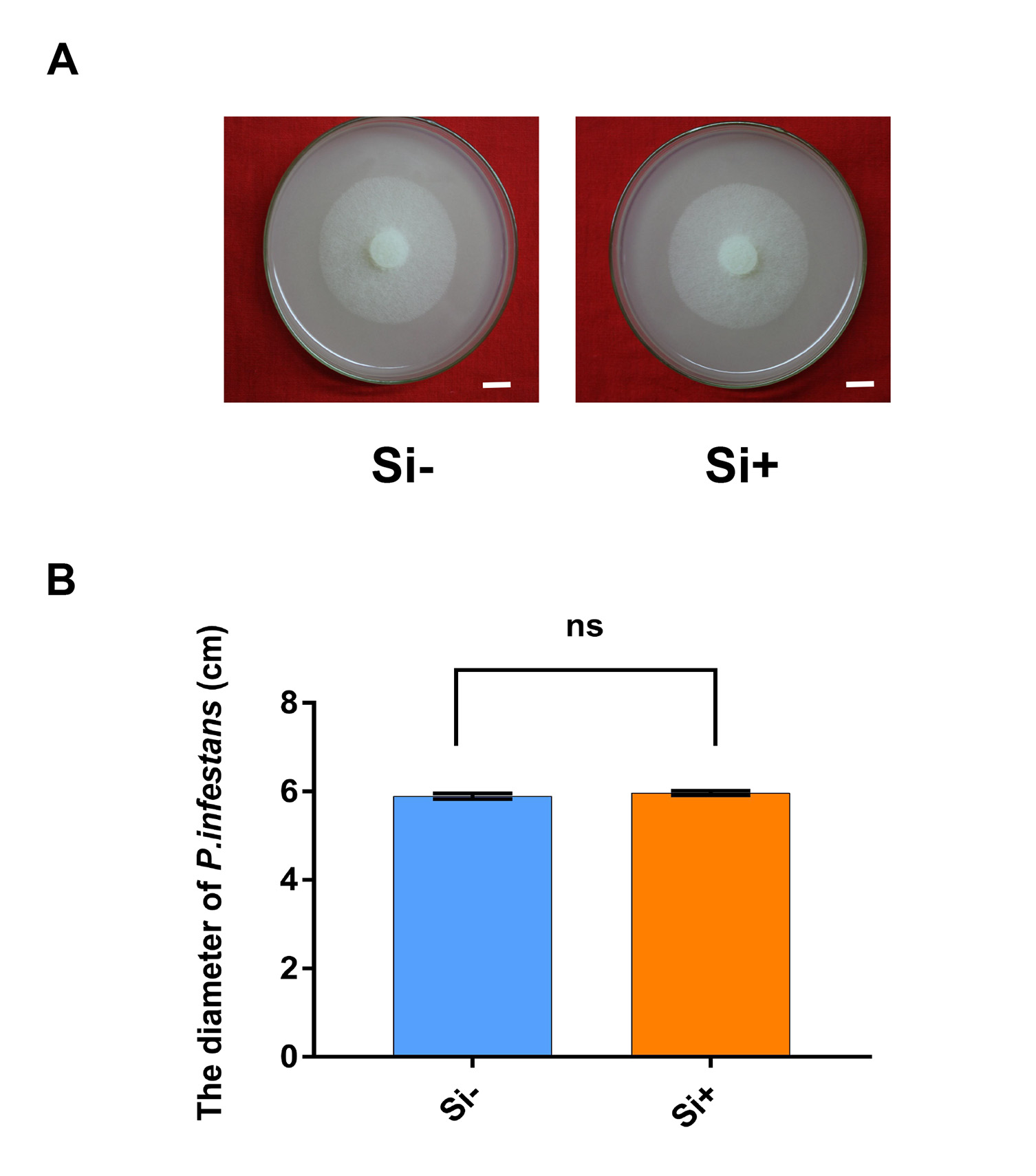

Supplement: Supplementary Figure 1 — Effects of Si on the growth of P. infestans in vitro. Scale bar represents 1 cm. (A) The growth of P. infestans EC1 on the Rye A medium (control) or Rye A containing 100 mM Na2SiO3. The images were photographed at 7 days. (B) The colony diameter of P. infestans EC1 in (A). Data represent the mean ± SD (n = 18). ns indicates no significant differences compared with control. [file Image_1.JPEG]

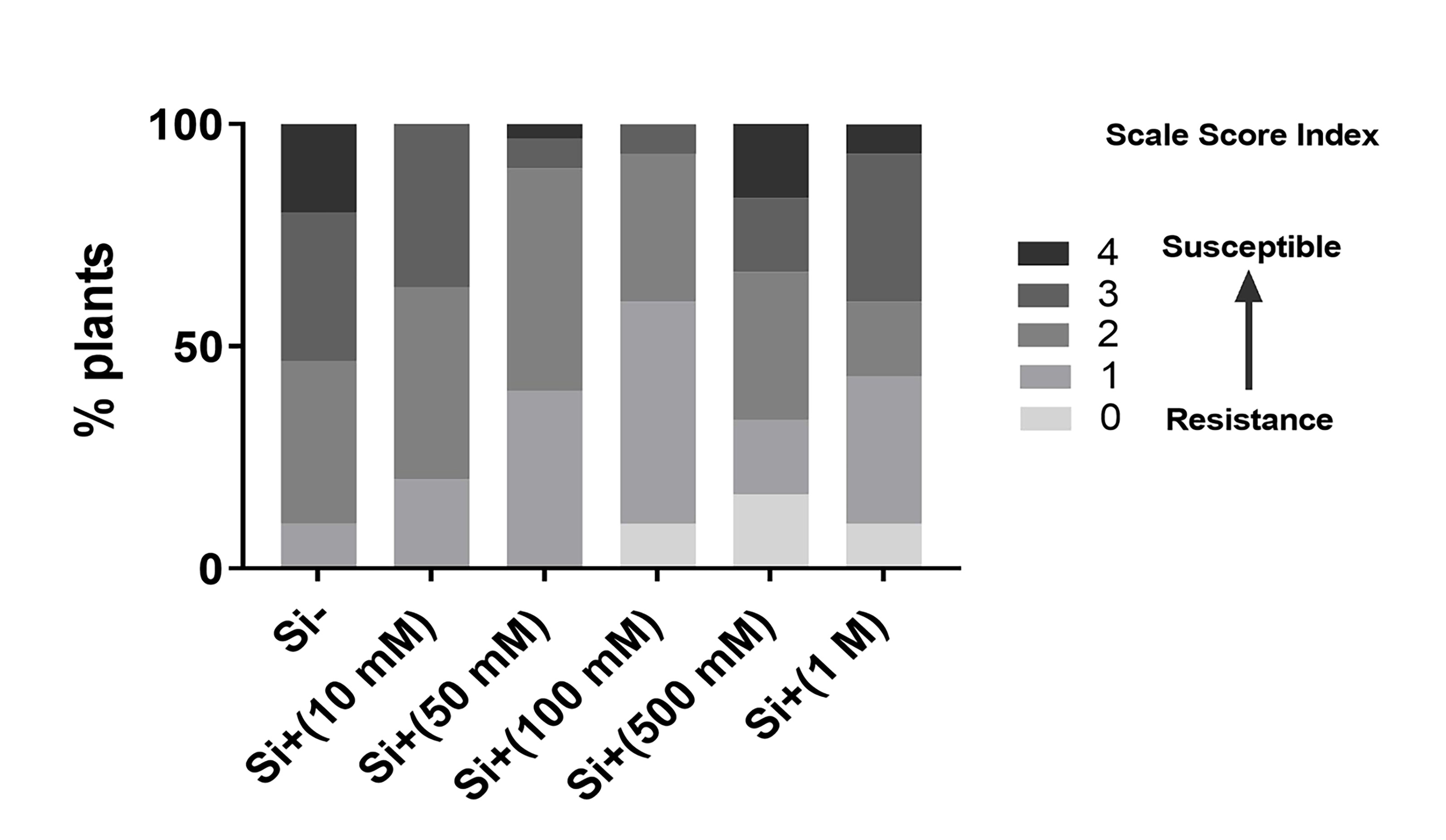

Supplement: Supplementary Figure 2 — The disease index of potato resistance to P. infestans after foliar spraying with 10, 50, 100, 500, or 1,000 mM Si. Over 30 leaves from 10 plants were scored for 0 to 4 levels. [file Image_2.JPEG]

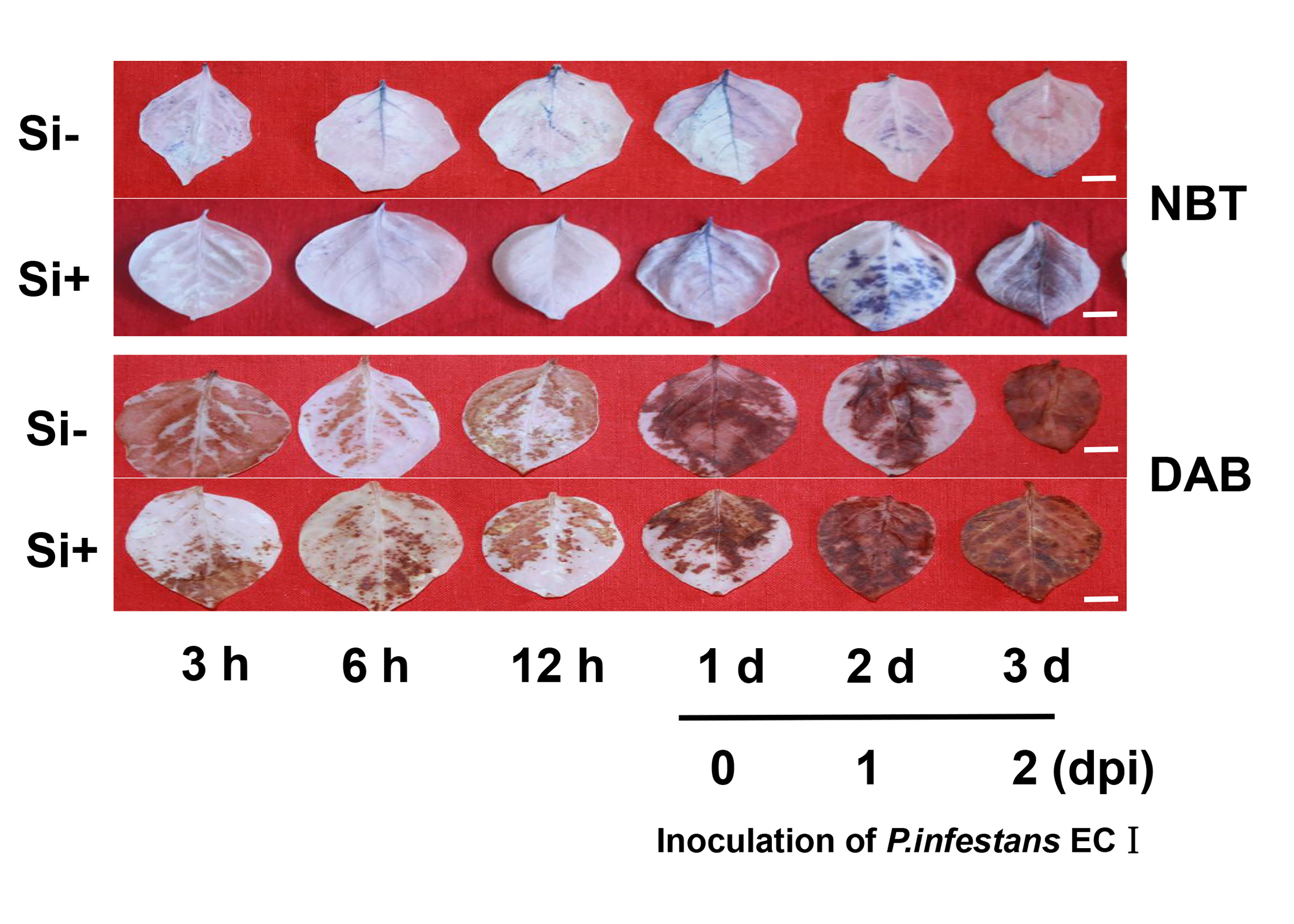

Supplement: Supplementary Figure 3 — Accumulation of ROS in the inoculated potato leaves in response to Si treatment. Potato leaves were stained with DAB and NBT to assess the accumulation of the hydrogen peroxide (H2O2) and superoxide (O2.–), respectively. Each three plants were sprayed with Si or control (Water), subsequently inoculated with P. infestans EC1 after 24 h. The experiments were repeated three times with similar results. Scale bar represents 1 cm. [file Image_3.JPEG]

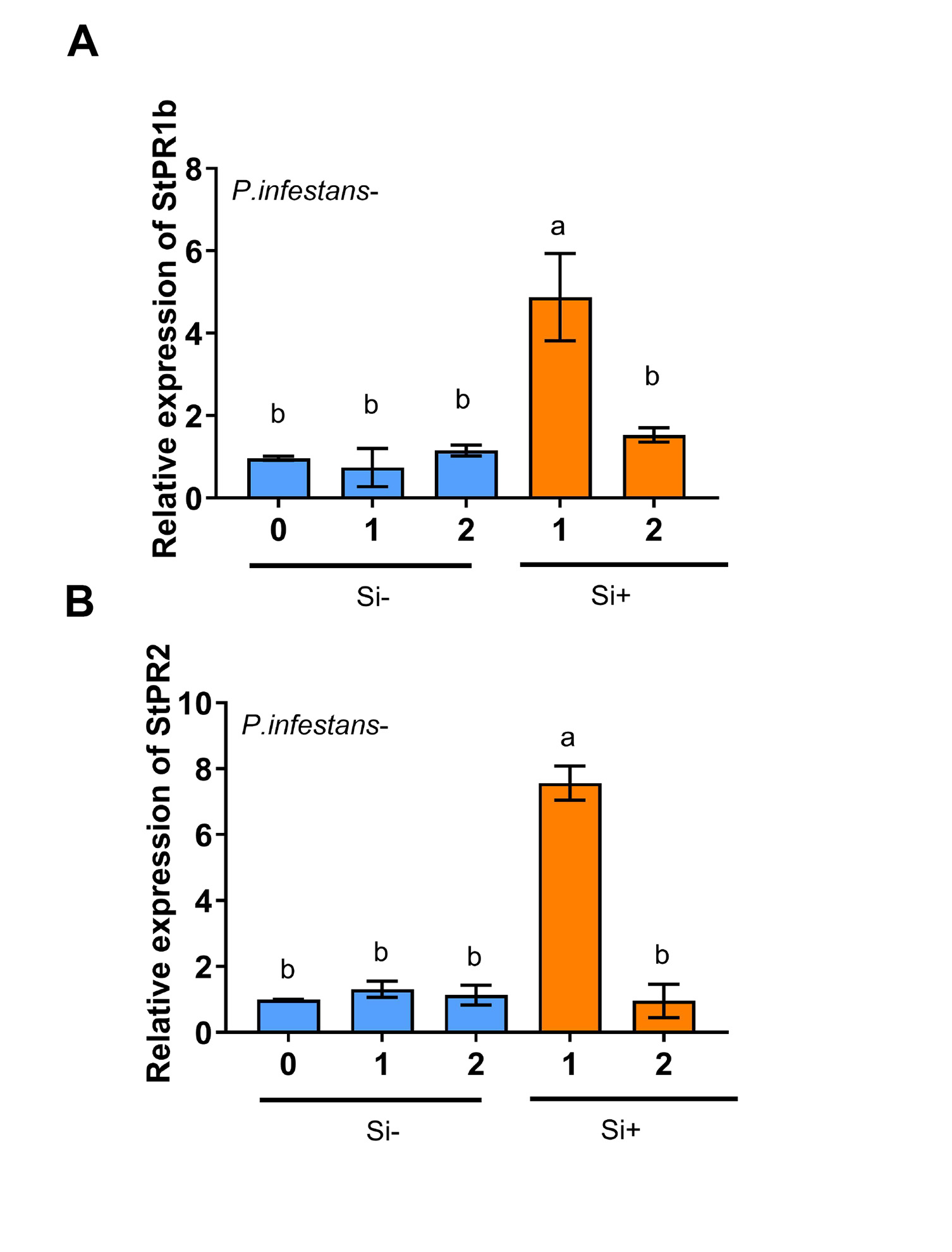

Supplement: Supplementary Figure 4 — The relative expression of StPR1b and StPR2 under foliar treatment of Si without EC1 inoculation. The expression of StPR1b (A) and StPR2 (B) with or without foliar treatment of Si. Potato leaves were treated with water or Si, and collected RNA samples at 0, 1, and 2 days. The StEF1 gene was used as a control to normalize expression. Letters above bars indicate significant difference among treatments (Tukey’s multiple range test, p < 0.05). Three biological repeats were performed with similar results. [file Image_4.JPEG]

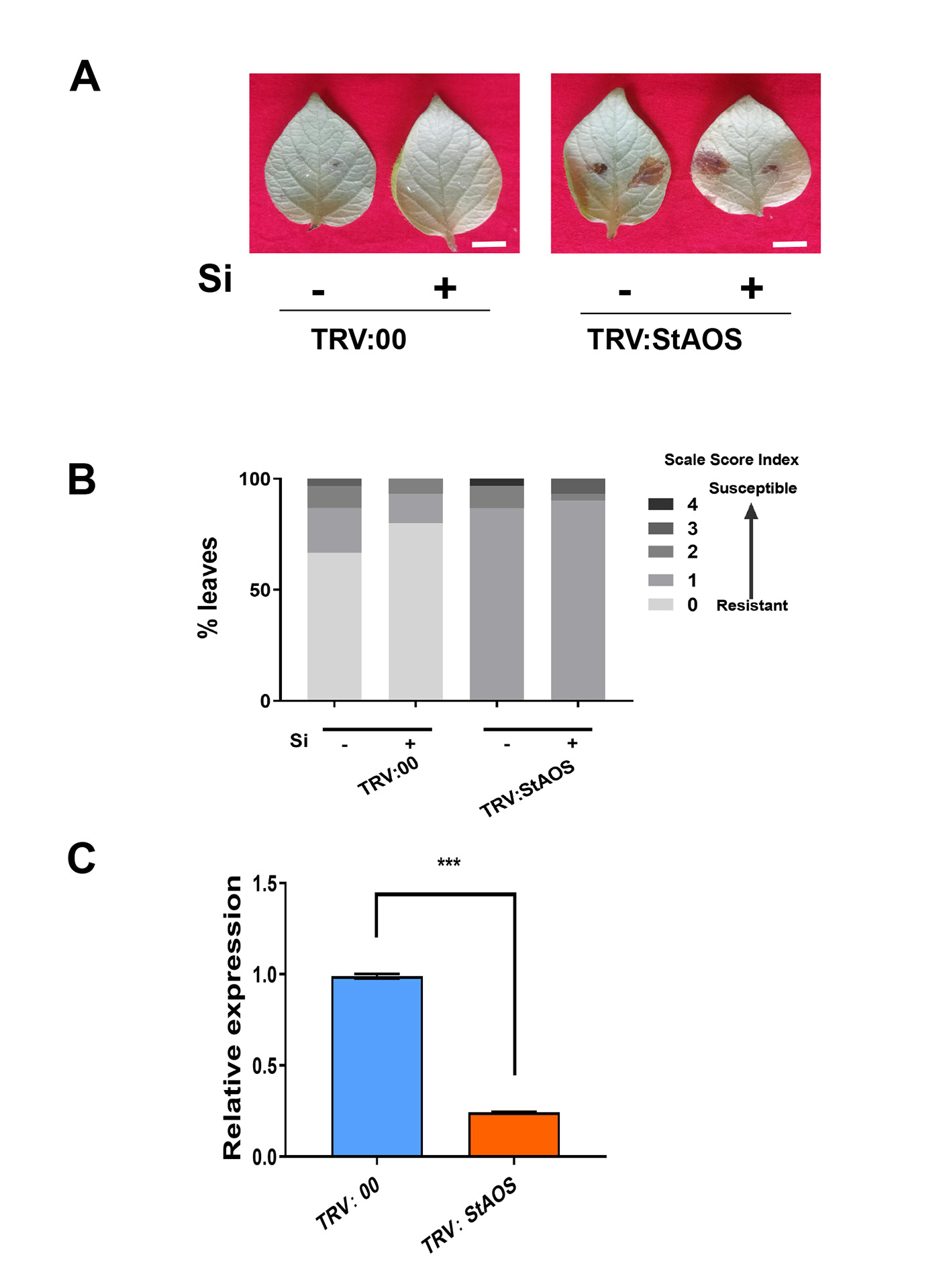

Supplement: Supplementary Figure 5 — The Si-mediated resistance was alleviated in StAOS-VIGS plants. Disease symptom (A) and disease index (B) in response to Si treatment. Scale bar represents 1 cm. Fifteen leaves from five individuals were inoculated with P. infestans EC1 at 24 h following Si treatment. The images and data were photographed and analyzed at 2.5 dpi, respectively. (C) The transcription level of StAOS were dramatically repressed in VIGS plants quantified by using qRT-PCR. The StEF1 gene was used as a control to normalize expression levels. ∗∗∗Indicates significant differences from control at P < 0.001. Triple biological repeats were performed with similar results. [file Image_5.JPEG]
