# Supplementary material for: Foliar Application of Silicon Enhances Resistance Against Phytophthora infestans Through the ET/JA- and NPR1- Dependent Signaling Pathways in Potato
Source: Front Plant Sci. 2021 Jan 28;12:609870. doi: 10.3389/fpls.2021.609870 (PMC7876464; doi:10.3389/fpls.2021.609870)
Supplement: Supplementary Table 2 — List of oligonucleotide primers used in this study. [file Table_2.doc]

Supplemental Table S2. List of oligonucleotide primers used in this study.

| **Primers** | **Sequence shown in 5’→3’ orientation** | **Description** |
| --- | --- | --- |
| StEF1-F | CAAGGATGACCCAGCCAAG | qRT-PCR primers for StEF1α (LOC102600107) |
| StEF1-R | TTCCTTACCTGAACGCCTGT |
| PiO8-F | GAAAGGCATAGAAGGTAGA | qRT-PCR primers for PiO8 |
| PiO8-R | TAACCGACCAAGTAGTAAA |
| StACS3F | CGAAAAGGGCATCCACCTCA | qRT-PCR primers for StACS3 (LOC102583502) |
| StACS3R | ATCGCACCAATGCGAAATCC |
| StACS2F | GTTGGATGGATTTGCGTCCG | qRT-PCR primers for StACS2(LOC102577574) |
| StACS2R | CCACCCTGGCTCTTGACATT |
| StEIN2F | AATGTCCCTGTAGCCCGAAA | qRT-PCR primers for StEIN2 (LOC102594025) |
| StEIN2R | TCGACCACCAGATTGTTCGA |
| StEIN2(VIGS)F | CGGGATCCCTATGTTCTCAACCGTCTTCAGGG | 5’ primer of StEIN2 (LOC102594025) for VIGS; *Bam*H I site is underscored  3’ primer of StEIN2 (LOC102594025) for VIGS; *Kpn* I site is underscored |
| StEIN2(VIGS)R | GGGGTACCCGACAGGAAATTGCCGTCTCCAC |
| StETR1(VIGS)F | CGGGATCCCAGGCTGAAGATGATGAG | 5’ primer of StETR1 (LOC102588584) for VIGS; *Bam*H I site is underscored  3’ primer of StETR1 (LOC102588584) for VIGS; *Kpn* I site is underscored |
| StETR1(VIGS)R | GGGGTACCACTTCCTTCCACTATCCC |
| StETR1F | AGCAACTACAAATTCCGGCG | qRT-PCR primers for StETR1 (LOC102588584) |
| StETR1R | TTGCTGGCAATTTGGTCACA |
| StNPR1(VIGS)F | CGGGATCCGACAAGTTTCAGCGACACCTATT | 5’ primer of StNPR1 (LOC102592156) for VIGS; *Bam*H I site is underscored  3’ primer of StNPR1 (LOC102592156) for VIGS; *Kpn* I site is underscored |
| StNPR1(VIGS)R | GGGGTACCATCATCAGAGTCCAATGCCCTAT |
| StNPR1F | GCACTTGAATCGGCTTAGGG | qRT-PCR primers for StETR1 (LOC102592156) |
| StNPR1R | GCTTCTTCAGTTGACGCTCT |
| StOPR3(VIGS)F | CGGGATCCTCGGCCTTGAATGCTATTGAAGCAG | 5’ primer of StOPR3 (LOC102586989) for VIGS; *Bam*H I site is underscored  3’ primer of StOPR3 (LOC102586989) for VIGS; *Kpn* I site is underscored |
| StOPR3(VIGS)R | GGGGTACCGCTGGTGAAACTCTAACGCCTAC |
| StOPR3F | TGATTTCCCCGACTTCAGCT | qRT-PCR primers for StOPR3 (LOC102586989) |
| StOPR3R | CATGAGATGCACGACCAACA |
| StSID2F | TGATTTCCCCGACTTCAGCT | qRT-PCR primers for StSID2 (LOC102589572) |
| StSID2R | CATGAGATGCACGACCAACA |  |
| StSID2(VIGS)F | CGGGATCCTCCAGCAGTTTGTGGGTA | 5’ primer of StSID2 (LOC102589572) for VIGS; *Bam*H I site is underscored  3’ primer of StSID2 (LOC) for VIGS; *Kpn* I site is underscored |
| StSID2(VIGS)R | GGGGTACCAGGTGCCTCAAGTTTCAT |
| StCOI1F | GGGCACATGAACTAGCATCG | qRT-PCR primers for StCOI1(LOC102584101) |
| StCOI1R | TTAAATGCGCCACCACCAAA |
| StCOI1(VIGS)F | CGGGATCCTTACTGCAAAGGTGTCCCAACTTGG | 5’ primer of StSID2 (LOC102584101) for VIGS; BamH I site is underscored  3’ primer of StSID2 (LOC102584101) for VIGS; Kpn I site is underscored |
| StCOI1(VIGS)R | GGGGTACCGGACACCATTATCAAGTGGCAGA |
| StAOS(VIGS)F | CGGGATCCTTCAGAACGAACATGCCACC | 5’ primer of StAOS (GenBank: ABD15175.1) for VIGS; *Bam*H I site is underscored  3’ primer of StAOS (GenBank: ABD15175.1) for VIGS; *Kpn* I site is underscored |
| StAOS(VIGS)R | GGGGTACCACGGCGAGAAGAAAGAAGGA |
| StAOSF | TTGAAACCCTAGATAAGGAAATGGC | qRT-PCR primers for StAOS(GenBank: ABD15175.1) |
| StAOSR | AAGCCCCCAACGCCGACTTATCAA |
| StERF1 | TGCGCGTGCATTGTGTAAAA | qRT-PCR primers for StERF1(LOC102590684) |
| StERF1 | CCACCACAGAAGACGACGAA |
| StACS1 | GACGCTCATGTTTTGCCTCG | qRT-PCR primers for StACS1(GenBank: BAB20862.1) |
| StACS1 | CTATTTCAGCCCCGGTCCTC |
| StACS7 | TCCCTGGATTTAGGGTTGGGA | qRT-PCR primers for StACS7(LOC102605100) |
| StACS7 | AATTTCATCGCGCTCTTCGC |
| StACS9 | TCCATTTTGTGCAACTGATGAAAGA | qRT-PCR primers for  StACS9(LOC102582710) |
| StACS9 | ACAAATTCCACAGTGAATCCCAC |
| StPDF1.2F | TCTTTTGCCTCGTCCTTGTT | qRT-PCR primers for StPDF1.2(LOC102591736) |
| StPDF1.2R | TTGTGACCCCATGGTTTGTA |
| StJR1F | CCATGATTATGGGACCGAAC | qRT-PCR primers for StJR1(LOC102583489) |
| StJR1R | AGCACGGGGTAACTTTTCCT |
| StLOXF | CAGATCAGGCCCCGTTAATG | qRT-PCR primers for StJR1(Y18548.1) |
| StLOXR | CCTGTAAGTCCACCTTCACTTGTTG |
| StPAL2F | GGTCACTGCCTCGGGTGAT | qRT-PCR primers for StJR1(X63104) |
| StPAL2R | CCTGCCAGTGAGCAAACCA |
| StPR2F | GTGAAGCTGGTTTGGGAAATG | qRT-PCR primers for StJR1(U019021) |
| StPR2R | TTGCCAATCAACGTCATGTCTAC |
| StPR1bF | GGCATCCCGAGCACAAAAT | qRT-PCR primers for StJR1(AY050221) |
| StPR1bR | CTGCACCGGAATCAAGT |
